# Turnip mosaic virus utilizes the lipid droplet biogenesis machinery to facilitate its propagation in plants

**DOI:** 10.1111/nph.71321

**Published:** 2026-06-09

**Authors:** Léna Jambou, Nathalie Arvy, Marguerite Batsale, Luc Sofer, Sara Shakir, Marielle Cochet, Vincent Simon, Charlotte Quinteau, Thomas Scotti, Nathan M. Doner, Satinder K. Gidda, Robert T. Mullen, Sabine D'Andréa, Jean‐Luc Gallois, Denis Coulon, Claire Bréhélin, Sylvie German‐Retana

**Affiliations:** ^1^ Laboratoire de Biogenèse Membranaire, UMR5200, CNRS University of Bordeaux Villenave d'Ornon F‐33140 France; ^2^ Biologie du Fruit et Pathologie, UMR 1332, Equipe de Virologie, INRAE University of Bordeaux Villenave d'Ornon F‐33882 France; ^3^ Department of Molecular and Cellular Biology University of Guelph Guelph N1G 2Z2 Canada; ^4^ Université Paris‐Saclay, INRAE, AgroParisTech Institute Jean‐Pierre Bourgin for Plant Sciences Versailles F‐78000 France; ^5^ INRAE, GAFL Montfavet F‐84143 France

**Keywords:** 6K2 vesicles, LDAP, lipid droplets, movement, neutral lipids, potyvirus, SEIPIN, viral replication compartment

## Abstract

- Lipid droplets (LDs), which are dedicated to storing neutral lipids (NLs), are dynamic organelles involved in numerous other functions, including membrane remodeling during abiotic stress. While the hijacking of LDs by animal viruses is well documented, their role(s) in plant virus infection remains largely unexplored. Similar to animal viruses, plant potyviruses reroute host proteins, intracellular membranes, and lipids to create an optimized microenvironment for their efficient viral replication compartment (VRC) assembly and movement. We therefore investigated whether potyviruses can also exploit the LD biogenesis machinery for their own benefit, akin to some animal viruses.
- Utilizing lipid analyses, confocal and transmission electron microscopy, and viral propagation surveys in plants impaired in LD biogenesis, we show that potyvirus turnip mosaic virus (TuMV) infection induces a significant accumulation of NLs and a proliferation of LDs in *Nicotiana benthamiana* or *Arabidopsis thaliana*‐infected leaves, which are often located in close proximity to VRCs. This suggests that there is a spatial and functional relationship between LDs and TuMV.
- We demonstrate also that two protein families crucial for LD biogenesis, namely the lipid droplet‐associated proteins and SEIPINs, facilitate TuMV propagation in *Arabidopsis thaliana*.
- Taken together, our results indicate a pro‐viral function for LDs in potyvirus‐infected plants.

## Graphical abstract

Potyvirus turnip mosaic virus induces neutral lipid accumulation and lipid droplet biogenesis in infected leaves for its own benefit.

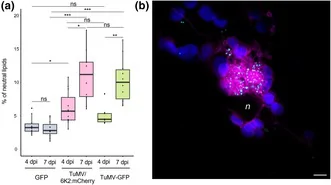

See also the Commentary on this article by May & Nagy, **252**: 525–527.

## Introduction

Lipid droplets (LDs) are ubiquitous organelles composed of a single phospholipid layer coated with proteins and enclosing a core of neutral lipids (NLs), mainly triacylglycerols (TAGs) and steryl‐esters (SEs) (Ischebeck *et al*., 2020; Farese & Walther, 2025). LDs are not, however, merely energy reservoirs involved in the storage and retrieval of lipids for energy homeostasis and membrane biogenesis; they are highly dynamic and can move through the cytoplasm, interact with other cellular organelles, and play various roles such as lipotoxicity prevention, signaling, and/or stress management (Bouchnak *et al*., 2023; Farese & Walther, 2025). The biogenesis of plant LDs is proposed to be similar to that in animal cells, whereby the synthesis and accumulation of TAGs and SEs occurs at endoplasmic reticulum (ER) membranes and that SEIPIN and other proteins regulate the filling, expansion, and budding of the nascent LD (Klug *et al*., 2024). Notably, the importance of SEIPINs in this process comes from observations that the overexpression of any of the three *SEIPIN* genes in Arabidopsis results in an increase in the size and number of LDs, whereas the disruption of *SEIPIN2* and *SEIPIN3* leads to the presence of fewer but aberrant‐sized LDs in seedlings (Cai *et al*., 2015; Taurino *et al*., 2018).

Plant LDs are primarily abundant in seeds, serving as energy reservoirs during postgermination growth. In vegetative tissues, such as in leaves, LDs are less prevalent but have diverse functions that are only beginning to be characterized (Bouchnak *et al*., 2023; Zhao *et al*., 2025). For instance, leaf LDs contribute to plant adaptation to abiotic stresses, including drought, heat, and cold, suggesting a functional shift from primarily lipid storage to more protective roles in response to environmental constraints (Gidda *et al*., 2016; Pyc *et al*., 2017; Huang, 2018). LD accumulation has also been observed in leaves and roots in response to fungal and bacterial infection (Schieferle *et al*., 2021; Scholz *et al*., 2025), suggesting that LDs may play a broader role in plant responses to biotic stresses. Leaf LDs are coated with various proteins (Horn *et al*., 2013; Cai & Horn, 2025), including the lipid droplet‐associated proteins (LDAPs), which function in LD size regulation (Gidda *et al*., 2016; Kim *et al*., 2016; Brocard *et al*., 2017). In addition, caleosins are also found on leaf LDs where they are considered to play a role in maintaining proper LD structure (Jiang *et al*., 2009; Liu *et al*., 2009; Brocard *et al*., 2017).

In animals, LDs are now recognized as active platforms for host defense against intracellular pathogens, such as bacteria and viruses (Bosch & Pol, 2022). Besides these potential defensive roles for LDs, their hyperaccumulation in animal cells is observed also following infection by protozoan parasites, bacteria and viruses, which are able to manipulate LDs to promote their replication, evade the immune system, and colonize the host (Safi *et al*., 2023). In particular, some positive‐stranded RNA viruses ((+) RNA viruses), which cause severe diseases in humans and animals, have been shown to hijack LDs (Hsia *et al*., 2024). These viruses recruit lipids and manipulate cell membranes to create membranous specialized compartments called viral replication compartments (VRCs). The VRCs provide a scaffold for the assembly of the replication machinery, including various viral and host‐cell factors, and protect double‐stranded RNA replicative forms against cellular defense mechanisms (Fernández de Castro *et al*., 2016). Notably, some animal (+) RNA viruses exploit LDs to generate VRCs (Laufman *et al*., 2019; Lee *et al*., 2019; Criglar *et al*., 2022) or to produce the energy needed to fuel the viral replication processes (Zhang *et al*., 2018).

Like their animal counterparts, plant (+) RNA viruses have the capacity to induce the proliferation of host‐cell membranes for the formation of VRCs, the structures that concentrate the viral replicase and host factors while shielding replication intermediates from host immune responses, such as RNA silencing (Laliberté & Zheng, 2014). Depending on the plant virus, VRCs are generated from various endomembranes, derived from either the ER, chloroplasts, mitochondria, or peroxisomes (Jin *et al*., 2018). Indirect links between LDs and plant virus infection have also been suggested in a few published studies, mainly those involving (+) RNA viruses of the *Tombusviridae* family (Chuang *et al*., 2014). Notably, in one such study describing ultrastructural changes induced by melon necrotic spot virus (MNSV) infection, the authors observed viral altered replication organelles frequently close to LDs (Gómez‐Aix *et al*., 2015), suggesting a possible role of LDs in the MNSV cycle. Recently, it has also been demonstrated that rice black‐streaked dwarf virus (RBSDV) infection in maize leads to elevated levels of polyunsaturated fatty acids (FAs) via stabilization of the LD‐associated protein ZmLDAP2, highlighting how viruses may exploit lipid remodeling to support replication (Wang *et al*., 2024). Additionally, the nucleocapsid protein encoded by *Valsa mali* negative‐strand RNA virus 1 (VmNSRV1), a mycovirus that can also spread in plant cells, was shown to be associated with and cause the structural rearrangement of LDs in both fungal and plant cells (Dai *et al*., 2024).

Besides the abovementioned studies concerning dsRNA or negative‐strand RNA phyto‐mycoviruses, no published data are available concerning any direct involvement of LDs in the infection cycle of plant (+) RNA viruses. Potyviruses represent one of the rare examples of plant (+) RNA viruses that utilize host endomembranes to produce membranous replication vesicles that are mobile between cells, reminiscent of that of animal viruses used to exit infected cells and enter into healthy cells (den Boon *et al*., 2010; Grangeon *et al*., 2013). Upon turnip mosaic virus (TuMV) and tobacco etch virus (TEV) infection, the viral membrane‐anchoring protein 6K2 (6 kDa protein2) induces the formation of ER‐derived replication vesicles that contain viral RNA as well as viral and host proteins involved in replication (Schaad *et al*., 1997; Beauchemin *et al*., 2007; Cotton *et al*., 2009). The ultrastructural characterization of TuMV‐induced cellular rearrangements showed the early formation of convoluted membranes connected to the ER in systemically infected leaves, followed by the production of single‐membrane vesicle‐like (VL) structures (Wan *et al*., 2015). Interestingly, these so‐called ‘electron dense bodies’ were found in the proximity of TuMV induced membranous replication compartments. Although these structures were not identified as LDs, their morphological characteristics based on electron microscopy studies strongly suggest they are LDs.

Given that like (+) RNA viruses in animals, (+) RNA plant viruses, especially potyviruses, hijack host proteins, intracellular membranes, and lipids to build an optimized lipid/membrane microenvironment for their efficient VRC assembly, we explored the possible involvement of LDs in plant virus infection. We first monitored the impact of TuMV infection on the leaf lipid metabolism and showed that TuMV‐infected leaves accumulate a higher amount of NLs and LDs than healthy leaves. We also observed by confocal and electron microscopy that the newly synthesized LDs are mostly located in proximity to the 6K2‐induced viral vesicles, and we demonstrated, using Arabidopsis mutants impaired in LD biogenesis, that LDs play a pro‐viral role in TuMV infection.

## Materials and Methods

### Plant materials, growth conditions

All the *Arabidopsis thaliana* lines (Arabidopsis) used in this study are in the Col‐0 background. Arabidopsis *ldap1* mutant is a T‐DNA insertion line (SALK_148081C) obtained from the Arabidopsis Biological Resource Center. The triple mutant *ldap1 ldap2 ldap3* was generated using CRISPR‐/Cas9‐based genome editing (Wang *et al*., 2015). Briefly, *ldap1* T‐DNA mutant plants (GABI‐Kat line GK‐309G05) were cotransformed via the floral dip method (Clough & Bent, 1998) with pBEE401E/LDAP2sgRNA1 or pBEE401E/LDAP2sgRNA2 and pBEE401E/LDAP3sgRNA1 or pBEE401E/LDAP3sgRNA2 binary plasmids encoding *CAS9* and single‐guide RNAs corresponding to sequences in the *LDAP2* and *LDAP3* open reading frames, respectively. Disruption of *LDAP1* expression in the triple mutant *ldap1 ldap2 ldap3* line was confirmed by real‐time polymerase chain reaction (RT‐PCR), and the disruption of expression of *LDAP2* and *LDAP3* transcripts (via the introduction of a premature stop codon in both ORFs) was confirmed by RT‐PCR and sequencing (Supporting Information Fig. S1).

The complemented mutant line, *ldap1*/*pLDAP1::LDAP1‐mCherry* (termed *LDAP1*_*pro*_ *:LDAP1*) corresponds to the *ldap1* mutant complemented with a mCherry fusion at the C‐ter of AtLDAP1 protein, expressed under the control of the native *AtLDAP1* promoter. The absence of endogenous LDAP1 and the presence of the LDAP1‐mCherry fusion protein in this line were confirmed by immunoblot analysis (Fig. S1). The overexpressing (OE) lines p35S::*LDAP1‐mCherry* (termed *LDAP1 OE*), p35S::SEIPIN1‐mCherry (termed *SEI1 OE*), and the double ko *seipin2 seipin3* are described in Gidda *et al*. (2016), Cai *et al*. (2015), and Greer *et al*. (2020), respectively.

### Molecular clones and fluorescent tagged viral infectious clones

The viral constructs pCambiaTuMV‐6K2‐mCherry (termed TuMV/6K2:mCherry), pCambiaTuMV/6K2:mCherry//GFP‐HDEL (termed TuMV/6K2:mCherry//GFP), the mutant pCambiaTuMV^W15A^/6K2:mCherry//GFP‐HDEL (termed TuMV^W15A^/6K2:mCherry//GFP), and the corresponding wild‐type (WT) construct pCambiaTuMV^WT^/6K2:mCherry//GFP‐HDEL (termed TuMV^WT^/6K2:mCherry//GFP) were kindly supplied by Dr J. F. Laliberté and described in Jiang *et al*. (2015). The pCBTuMV‐GFP//mCherry‐HDEL construct (termed TuMV‐GFP//mCherry) and pCamTuMV‐GFP (termed TuMV‐GFP) were kindly provided by Dr A. Wang (Dai *et al*., 2020).

pBEE401E/LDAP2sgRNA1, pBEE401E/LDAP2sgRNA2, pBEE401E/LDAP3sgRNA1, and pBEE401E/LDAP3sgRNA2 were constructed for CRISPR‐/Cas9‐based genome editing of Arabidopsis *LDAP2* and *LDAP3*, respectively, using the methods described by Wang *et al*. (2015). Primers corresponding to sgRNAs were designed using CRISPR‐PLANT v2 (http://www.genome.arizona.edu/crispr2/) (Minkenberg *et al*., 2019) to specifically target regions in either the *LDAP2* or *LDAP3* ORFs (Fig. S1). Cas9 and sgRNA primer sequences are shown in Table S1. Resulting PCR products were digested with *Bsa*I and then ligated into similarly digested pBEE401E (Wang *et al*., 2015), yielding pBEE401E/LDAP2sgRNA and pBEE401E/LDAP3sgRNA.

pLDAP1::LDAP1‐mCherry was constructed using Gateway cloning technology. PCR amplification of the *LDAP1* gene, including its promoter region up to −855 from the ATG, using attB1_pro_gene_LDAP1 and attB2_LDAP1_tag and the Q5 high‐fidelity DNA polymerase (NEB) was introduced in the pMDC140NoPro‐mCherry destination vector generated by (1) inserting the *mCherry* gene, PCR‐amplified using mCherry‐fwd and mCherry‐rev primers, between the *Asc*I and *Sac*I sites of pMDC140 (Curtis & Grossniklaus, 2003), and (2) removing the p35S promoter through *Hind*III and *Xba*I digestion followed by Klenow fill‐in and ligation. Primer sequences are shown in Table S1.

### Plant virus inoculation

The viral coding sequences were inserted in a binary vector which allows plant inoculation by agroinfiltration of the leaves and initial expression of the viral genome under the 35S promoter in the primary infected cells. As such, the virus can replicate and propagate from cell to cell in the inoculated leaf as well as in sink leaves through systemic movement (Vaghchhipawala & Mysore, 2008).

For agroinoculation of the viruses in *Nicotiana benthamiana*, *Agrobacterium tumefaciens* cells containing TuMV‐derived clones were infiltrated into the leaves at an OD_600_ of 0.03. For *Arabidopsis thaliana*, *A. tumefaciens* cells containing TuMV‐derived clones were infiltrated via a specific buffer (10 mM MES pH 5.6, 10 mM MgCl_2_, 150 μM acetosyringone) into the plant leaves at an OD_600_ between 0.5 and 0.6.

### Monitoring of TuMV‐green fluorescent protein (GFP) propagation

To monitor TuMV‐GFP propagation in the inoculated leaves, we optimized the OD_600_ values for agroinfiltration in order to monitor at 5 d post‐inoculation (dpi) individual nonconfluent infection foci (originating from single inoculated cells) under the Macroscope ZEISS AxioZoom. Pictures were taken at ×40 magnification. The area of the infection foci was measured using imagej software (Schneider *et al*., 2012) and normalized relative to the mean value of infection foci measured on Col‐0 and set at 100%.

### Confocal microscopy analyses and quantification of LDs

All confocal observations were performed at the same time of day (i.e. in the morning) to ensure comparability across samples. Monodansylpentane (MDH) was used as a blue‐fluorescent marker for LDs (Yang *et al*., 2012). The staining was made by infiltration of the leaves with MDH solution (0.2 mM in phosphate‐buffered saline). MDH, GFP, and mCherry were excited at 405, 488, and 561 nm, respectively, and their emission light captured at 418–482, 499–544, and 579–633 nm, respectively. To quantify the number of LDs μm^−2^, the area on the epidermal cell plan was measured with polygon selection tool. In this area, the multi‐point tool on imagej/fiji was used to count LDs.

### Transmission electron microscopy (TEM)

*Nicotiana benthamiana* leaves were cut into 2 mm size squares, chemically fixed and included in SPURR (EMS) resin according to Brocard *et al*. (2017). Ultrathin sections of 80 nm thickness were obtained with ultramicrotome EM UC7 (Leica). The transmission electron microscope Tecnai G2 Spirit TWIN 120 kV (FEI) equipped with a CCD 16Mpixels Eagle 4k camera was used for acquisition at 120 kV. ImageJ software was used for image analysis.

### Fluorometric camera analysis

Monitoring of TuMV‐GFP//mCherry infection in the systemically infected leaves of Arabidopsis was carried out by the use of a closed fluorometric camera FluorCam FC 800‐C/1010‐GFP (Photon System Instruments, Drasov, Czech Republic) equipped with a GFP filter, with emission detected either in the chlorophyll (Chl) fluorescence range (650–800 nm, red excited) or at the GFP emission peak (508 nm, blue excited), as described in Zafirov *et al*. (2021). Total Chl fluorescence was used to determine the total visible leaf area, which was then used to calculate the percentage of plant tissue exhibiting GFP fluorescence as estimated using fluorcam software.

### Lipid analysis

Total lipids from leaves were extracted by the Folch method (Folch *et al*., 1957) after phospholipase D inactivation using hot isopropanol, as described in Coulon *et al*. (2020). Neutral and polar lipids were separated by solid phase extraction (SPE) using a 1 ml SPE column (Interchim). NLs were eluted using 1 ml hexane : diethyl ether (4 : 1 v/v), pigments with 1 ml hexane : diethyl ether (1 : 1 v/v), and polar lipids with 1 ml methanol and 1 ml chloroform. Lipid‐derived fatty acids were transesterified and quantified by Gas Chromatography (GC) (Agilent 7890 gas chromatograph equipped with a Carbowax column, Alltech Associates, Deerfield, IL, USA) coupled to a flame ionization detector (FID) as described in Coulon *et al*. (2020) using heptadecanoic‐acid methyl ester as standard.

### RNA extraction and quantitative RT‐PCR

Total RNA extractions were performed using the NucleoSpin® RNA Plant Kit (Macherey Nagel, SAS, Hoerdt, France) as described in Xue *et al*. (2024). Five independent plants for each treatment were sampled in 3 independent experiments. The purified RNAs were treated by Dnase I with the TURBO DNASE‐free TM Kit (Invitrogen, Thermo Fisher Scientific, Waltham, MA, USA), and RNA concentration was determined by measuring absorbance at 230, 260, and 280 nm. RNA was reverse transcribed using the RevertAid EP0442 enzyme (Thermo Scientific) and oligo (dT) (18) primer. Real‐time quantitative RT‐PCR was performed on the Light Cycler 480 Instrument II (Roche), using the Light Cycler®480 SYBR Green I MASTER Kit (Roche Diagnostics, Meylan, France). Primer sequences are provided in Table S1. The relative expression level of the target genes (CP, mCherry, or GFP fold changes) was expressed according to the reference gene (*Ubiquitin*) as 2^‐∆ (Ct target gene‐CtUb)^. Then, ratios of CP on mCherry or GFP expression transcripts transcribed from the cassette of the same construct but independent of the TuMV were calculated in order to avoid the bias of agroinfiltration efficiency.

To quantify the expression levels of *LDAP1* at 0, 5, and 21 dpi, four plants for each treatment at the three time points were sampled in three independent experiments. qRT‐PCR was performed using the primers qLDAP1‐Fwd and qLDAP1‐Rev (Table S1) designed and tested to amplify specifically *LDAP1*. The infection of TuMV in both inoculated leaves and systemically infected leaves was confirmed by qRT‐PCR with CP primers. The accumulation of TuMV genomic RNA in the inoculated leaves was quantified at 5 dpi relative to the number of infection foci by monitoring the CP/mCherry ratio (2^−Δ(CtCP–CtUb)^/2^−Δ(CtmCherry–CtUb)^). TuMV RNA accumulation in systemic leaves was quantified on pools of eight leaves randomly collected at 21 dpi from 15 plants of each genotype and expressed relative to the *Ubiquitin* reference gene (2^−Δ(CtCP–CtUb)^).

To quantify TuMV titer in virus‐induced gene silencing (VIGS) assay in *N. benthamiana*, the quality of RNA and a complete absence of DNA were verified by PCR using primers designed on the intron flanking region of *NbPP2A* (Niben101Scf11130g00001) (Table S1). The qRT‐PCR was performed for quantification of *NbLDAP1* and TuMV RNA as described above. Ct values of TuMV *CP* and the reference gene *EF1a* were obtained from four biological replicates with three technical replicates per sample. Mean Ct values were used to calculate ΔCt as (CtTuMV − CtEF1a). ΔΔCt values were determined by normalizing ΔCt values of each sample to those of the nontarget GUS (β‐glucuronidase) control. Relative fold change in TuMV *CP* expression was calculated using the 2^−ΔΔCt^ method.

### Protein extraction and immunoblot analysis

Total proteins were extracted according to Rensink *et al*. (1998) and concentrated by chloroform–methanol precipitation (Wessel & Flügge, 1984). Fifty micrograms of total proteins were loaded and separated by SDS‐PAGE and transferred onto PVDF membranes. LDAP1 protein was detected using a specific serum (Coulon *et al*., 2024). Band intensities were quantified using image lab software (v.5.2; Bio‐Rad). Three independent biological replicates were used for quantification.

### VIGS assay

The vectors used for VIGS have been described previously (Liu *et al*., 2002). To identify the *LDAP1* ortholog in *N. benthamiana, LDAP1* orthologs of tomato (Solyc 05g015390) and *A. thaliana* (At1g67360) were used as reference sequences on Solgenomics and www.nbenth.com websites and we found the only single best hit in the *N. benthamiana* genome (*NbLDAP1*, Nbv6.1TrP68607), showing 54–83% identity, respectively (Table S2). A 300 bp fragment of *NbLDAP1* cDNA (nt 468–767) was identified as the best target using the VIGS tool on Solgenomics and amplified by PCR using primers described in Table S1. The amplified fragment was transferred into pTRV2–pYL276 through the Gateway cloning technology, resulting in pTRV2‐*NbLDAP1*. VIGS assays were performed as described in Senthil‐Kumar & Mysore (2014) with slight modifications. Briefly, *A. tumefaciens* carrying pTRV1 and pTRV2‐*NbLDAP1* or pTRV2‐GUS were mixed with OD_600_ of 0.3 each and inoculated into 4‐wk‐old *N. benthamiana* plants. As a visual control, pTRV1 mixed with pTRV2‐*NbPDS* were used. Two weeks later, upon leaf bleaching in pTRV2‐*NbPDS* control plants, fully expanded young leaves plants were infiltrated with *A. tumefaciens* carrying the TuMV‐GFP infectious clone. For TuMV local infection assays, samples were collected at 6 dpi to detect viral accumulation and for TuMV systemic infection assays, three most apical leaves of each plant were collected at 2 week post‐inoculation to detect viral accumulation.

### Statistical analysis

After verifying the normal distribution of the data, differences between the various measured parameters were analyzed using Student's *t*‐test for normally distributed data or the Kruskal–Wallis test followed by Dunn's *post hoc* test or Wilcoxon's test for nonnormally distributed data. All statistical tests were performed using R software (R Core Team, 2025). The degree of significance is indicated by asterisks according to the *P*‐values (*, *P* < 0.05; **, *P* < 0.01; ***, *P* < 0.001).

## Results

### TuMV infection induces the accumulation of NLs in infected leaves of *N. benthamiana*

**Fig. 1:**
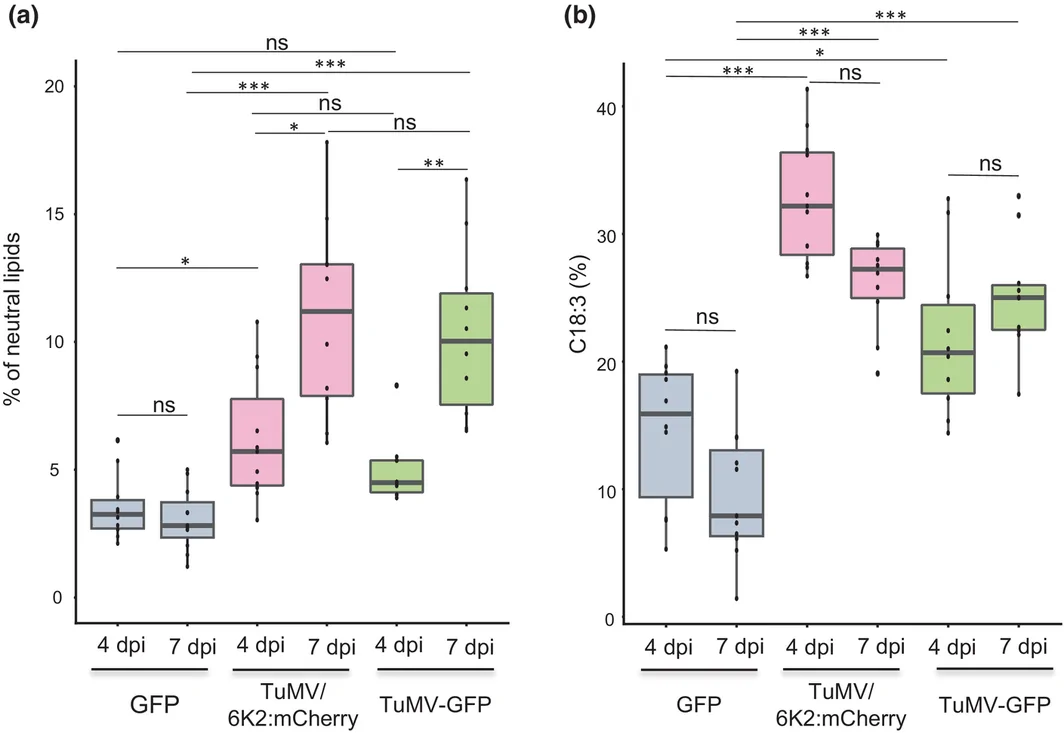
Turnip mosaic virus (TuMV) infection induces an increase of neutral lipids (NLs) content in *Nicotiana benthamiana* leaves. (a) NL content and (b) C18:3 content in NLs of *N. benthamiana* leaves agro‐inoculated with a mock control expressing a free green fluorescent protein (GFP), or viral constructs TuMV/6K2:mCherry and TuMV‐GFP. Lipids from inoculated leaves (4 d post‐inoculation (dpi)) or from systemically infected leaves (7 dpi) were extracted. NL and C18:3 contents were determined by GC‐FID after solid phase extraction (SPE) separation. These data combined 12 biological replicates from two independent experiments. Thick horizontal lines within the boxes represent the median of the measurements. Box plots display the median (thick horizontal line), interquartile range (IQR, box), and whiskers extending to 1.5 × IQR (25^th^ percentile) and Q3 (75^th^ percentile). Points indicate the value of each replicate. Points beyond the whiskers are considered as outliers. *, *P* < 0.05; **, *P* < 0.01; ***, *P*<0.001 (Kruskal–Wallis followed by Dunn's *post‐hoc*). ns, not significant.

To determine how TuMV infection impacts lipid metabolism in leaves, and in particular the biosynthesis of NLs that accumulate mainly in LDs, *N. benthamiana* plants were inoculated with two different TuMV infectious clones used throughout this study: TuMV‐GFP (Dai *et al*., 2020) and TuMV/6K2:mCherry (Jiang *et al*., 2015), containing sequences encoding the GFP within the TuMV genome or encoding a 6K2:mCherry fusion protein, respectively. The presence of the virus in the inoculated and systemically infected leaves was estimated by observation of the GFP or mCherry fluorescence. Total lipids were extracted from the fluorescent areas sampled at 4 dpi for the inoculated leaves and 7 dpi for the systemically infected leaves. The ‘mock’ control corresponded to plants agroinfiltrated with a binary vector allowing the expression of GFP alone, which was driven by the 35S promoter. Neutral and polar lipids were separated by solid‐phase extraction and quantified by gas chromatography. A 2‐fold to 3.6‐fold increase in NL content compared to mock‐treated leaves was observed in inoculated and systemically infected leaves, respectively (Fig. 1a, for both viruses). Notably, viral infection led to a significant enrichment of the polyunsaturated C18:3 fatty acid in NLs compared to mock conditions. Indeed, at 7 dpi, the C18:3 content was 3.3‐fold and 3.1‐fold higher in NLs induced by TuMV/6K2:mCherry and TuMV‐GFP, respectively, relative to mock‐treated samples (Fig. 1b).

### TuMV infection induces LD accumulation in close proximity to VRCs in infected leaves of *N. benthamiana*

To establish whether the increase in NL amount caused by TuMV infection resulted in a modulation of LD abundance, we characterized LD dynamics in TuMV‐infected *N. benthamiana* leaves by laser‐scanning confocal microscopy at different dpi, in both inoculated and systemically infected leaves. *Nicotiana benthamiana* plants were inoculated with the TuMV/6K2:mCherry virus that allows visualization in infected cells of the TuMV‐produced 6K2‐mCherry vesicles labeling the VRCs near the nucleus. In addition, LDs were stained with the LD‐specific, cyan fluorescent dye MDH.

**Fig. 2:**
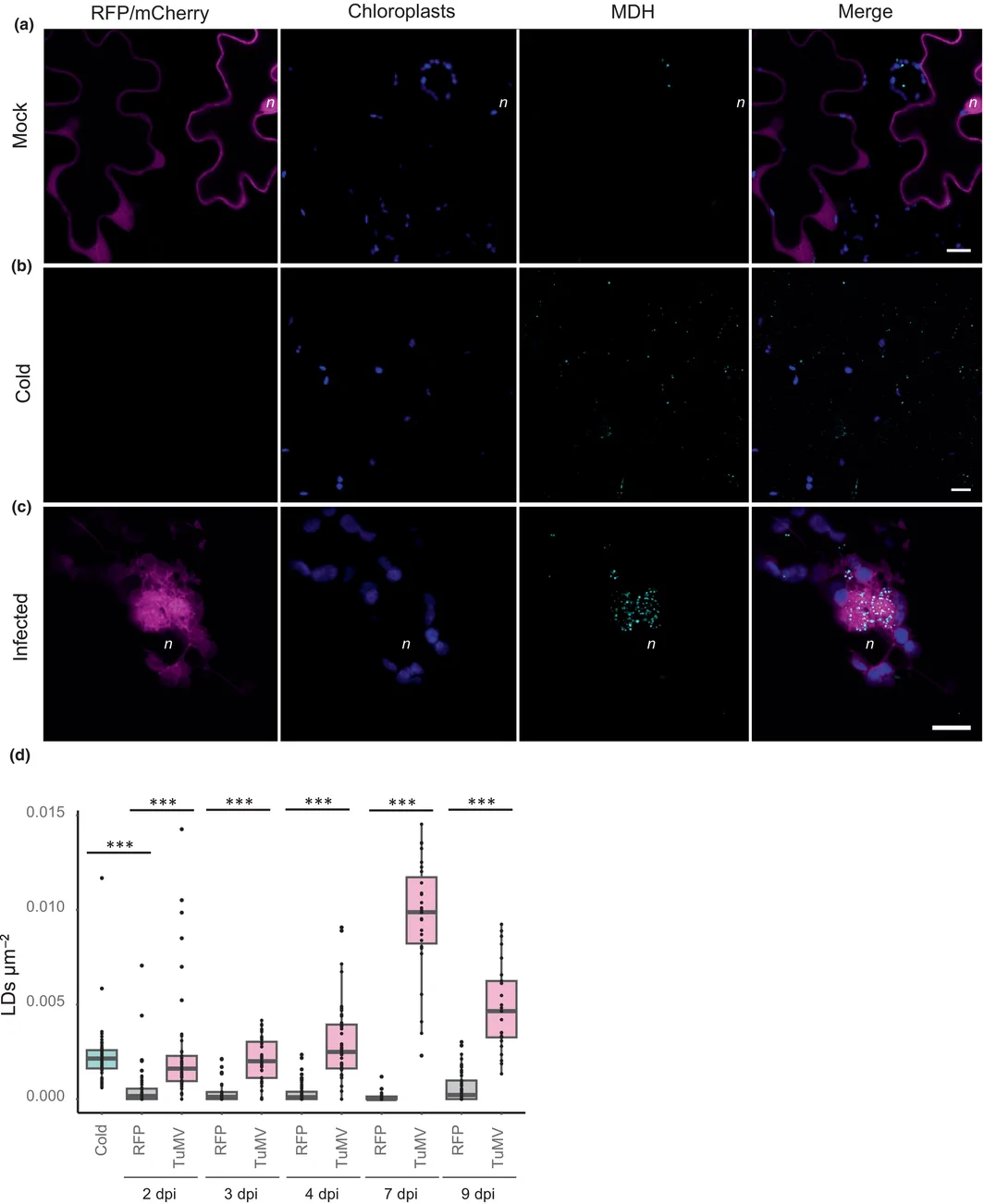
Observation by confocal microscopy of lipid droplet (LD) accumulation in *Nicotiana benthamiana* turnip mosaic virus (TuMV)‐infected leaves. *Nicotiana benthamiana* epidermal cells expressing free red fluorescent protein (RFP) (a), submitted to 4°C during 24 h (b), or inoculated with TuMV/6K2:mCherry construct at 4 d post‐inoculation (dpi) (c) were imaged by confocal microscopy after staining with the lipid droplet (LD)‐specific dye monodensylpentane (MDH). Fluorescence of RFP or 6K2:mcherry (magenta), LDs (cyan), and autofluorescence of Chl (dark blue) are visualized. Bars, 10 μm. *n*, nucleus. Images are representative of three independent experiments and at least three plants per experiment. (d) LD abundance (LDs per cell surface in μm^2^) in leaves of plants submitted to 4°C for 24 h (cold, in blue), expressing free RFP (RFP, in gray) or infected with TuMV/6K2:mCherry (TuMV, in pink) was quantified in inoculated leaves at 2, 3, and 4 dpi and in systemically infected leaves at 7 and 9 dpi. These data combined measures from three independent experiments, with 25–72 images per conditions analyzed. Box plots display the median (thick line), interquartile range (IQR, box), and whiskers extending to 1.5 × IQR from Q1 (25^th^ percentile) and Q3 (75^th^ percentile). Points indicate the value of each replicate. Points beyond the whiskers are considered as outliers. ***, *P* < 0.001 (Kruskal–Wallis followed by Dunn's *post‐hoc*).

For the mock condition, *N. benthamiana* plants were agroinfiltrated with a construct transiently expressing the red fluorescent protein (RFP) alone via the 35S promoter (Fig. 2a). In addition, as a control for LD biogenesis, *N. benthamiana* plants were subjected to cold stress at 4°C for 24 h (Fig. 2b), which causes LD proliferation in plant cells (Gidda *et al*., 2016). Upon infection by TuMV/6K2:mCherry, we observed numerous LDs that were clustered and localized in proximity to 6K2‐labeled VRCs near the nucleus (Fig. 2c), in both inoculated and systemically infected leaves. No similar LD aggregation was apparent in leaves expressing RFP alone (Fig. 2a) or subjected to cold stress (Fig. 2b), whereby LDs were more uniformly distributed throughout the cytoplasm. These results suggest that LDs are formed and/or recruited in the vicinity of the 6K2‐induced VRCs. Similar results were obtained with TuMV‐GFP in *N. benthamiana* leaves (Fig. S2). We next quantified the LD abundancy in cold‐stressed, mock‐inoculated or TuMV/6K2:mCherry‐infected leaves at 2, 3, and 4 dpi in inoculated leaves, as well as at 7 and 9 dpi in systemically infected ones, and observed a significant increase in LD abundancy in TuMV‐infected leaves (Fig. 2d) compared to the mock condition. Indeed, there were 6 and 78 times more LDs μm^−2^ at 4 dpi (3 × 10^−3^ μm^−2^ ± 2.1 × 10^−3^ vs 3.4 × 10^−4^ ± 5.6 × 10^−4^) and 7 dpi (9.6 × 10^−3^ μm^−2^ ± 3.1 × 10^−3^ vs 1.2 × 10^−4^ ± 2.6 × 10^−4^), respectively, in TuMV/6K2:mCherry‐inoculated leaves than in the mock‐inoculated ones. This increase was more pronounced in the TuMV/6K2:mCherry condition than in the cold stress condition (i.e. 4 times more at 7 dpi, with 2.3 × 10^−3^ μm^−2^ ± 1.5 × 10^−3^ in cold‐treated leaves).

**Fig. 3:**
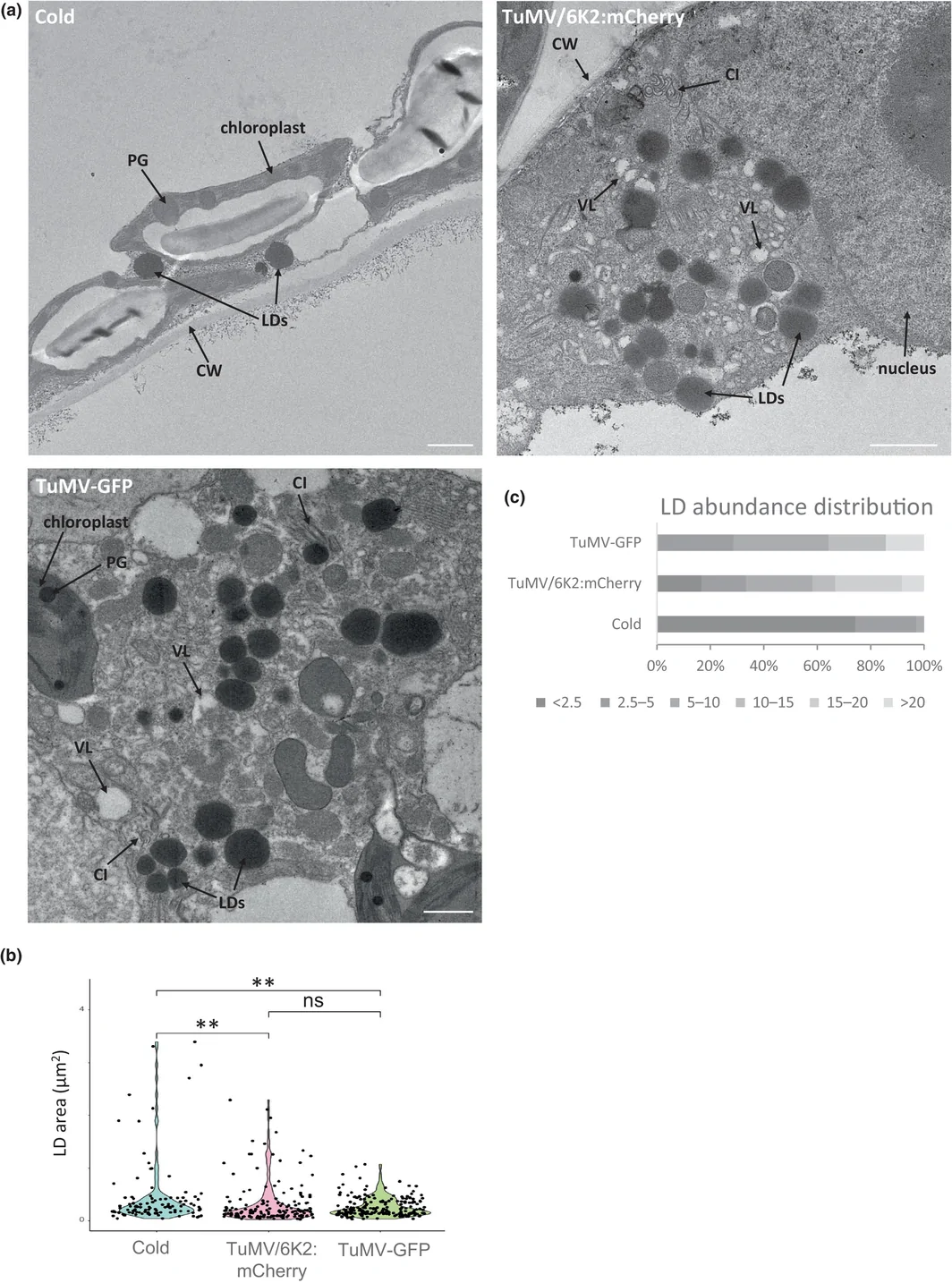
Lipid droplets (LDs) accumulate in close proximity to viral replication compartments (VRCs) in *Nicotiana benthamiana* turnip mosaic virus (TuMV)‐infected leaves. (a) Ultrastructure of parenchyma cells of *N. benthamiana* leaves subjected to 4°C during 24 h (cold), inoculated with TuMV/6K2:mCherry or systemically infected with TuMV‐green fluorescent protein (GFP) virus at 6 d post‐inoculation was observed by transmission electron microscopy. Numerous LDs are observed in the vicinity of viral vesicle‐like structures with both viruses. CW, cell wall; PG, plastoglobule; CI, cylindrical inclusion; VL, viral vesicle‐like structures. Bars, 1 μm. Images are representative of three different plants for each condition, with at least 35 cells observed per condition. (b) LD size and (c) distribution of LD number for each condition. LD area (μm^2^) and LD number per picture of 183 μm^2^ area were determined thanks to the ImageJ software. **, *P* < 0.01, as determined by Wilcoxon test; 110 < *n* < 203 for the determination of LD size, and 15 < *n* < 38 for LD number. ns, not significant.

We next performed TEM observations of TuMV/6K2:mCherry systemically infected leaves or TuMV‐GFP‐inoculated leaves, chemically fixed at 6 dpi. The observation of pinwheel‐shaped cylindrical inclusions (CI) in the cytosol (Fig. 3a), which are typical for infections with potyviruses (Sorel *et al*., 2014), confirmed the presence of TuMV in leaves. We also observed a multitude of single membrane VL structures ranging in size from 100 to 450 nm, comparable to those described by Wan *et al*. (2015) in the cytoplasm of TuMV‐infected cells. Among these numerous VL structures, LDs were present, implying that they were recruited to TuMV‐induced replication vesicles. The relatively close proximity of LDs and VL structures was frequently observed, which could allow the formation of membrane contact sites between both compartments (Fig. 3a). The average size of LDs was slightly bigger in cold‐treated leaves than in TuMV‐infected leaves (Fig. 3b), but LD sizes were less homogeneous in cold‐treated leaves than in virus‐infected ones, suggesting a tighter regulation of LD size in response to viral infection. While a maximum of six LDs was observed in a picture of 183 μm^2^ of a cold‐treated leaf cell section, up to 27 LDs were counted in a cell section of the same size from TuMV‐infected cells in proximity to VL structures, confirming the aggregation of LDs at 6K2‐induced VRCs in infected leaves (Fig. 3c).

### Silencing of *NbLDAP1* significantly impairs TuMV accumulation

**Fig. 4:**
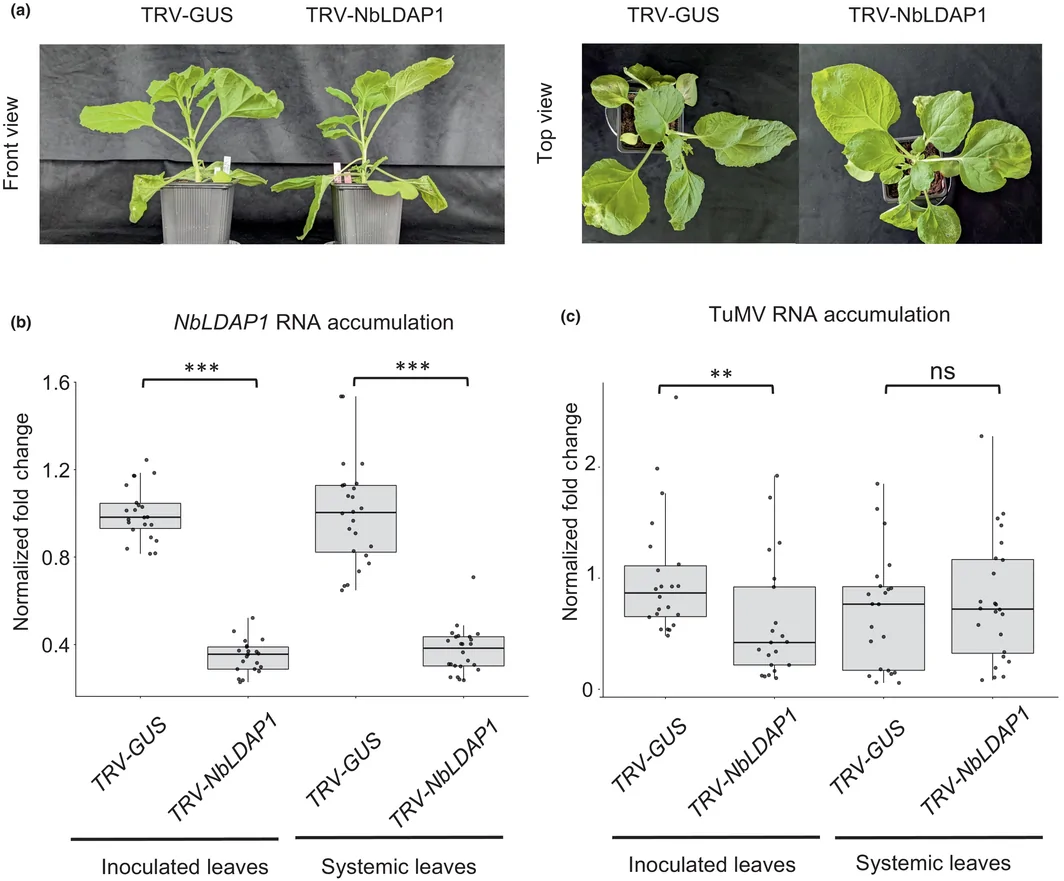
Silencing of *NbLDAP1* significantly reduces turnip mosaic virus (TuMV) accumulation in *Nicotiana benthamiana* leaves. (a) Representative pictures of *N. benthamiana* plants infected with the indicated combinations of viruses, at 14 dpi. (b) *NbLDAP1* RNA accumulation in local and systemic infections in control (TRV*‐GUS*) or in *NbLDAP1*‐silenced (TRV*‐NbLDAP1*) plants, measured by quantitative real‐time polymerase chain reaction (qRT‐PCR) using a primer pair targeting the *NbLDAP1* gene. The accumulation of *NbLDAP1* RNA is normalized to the *EF1a* reference gene. (c) Viral (TuMV) RNA accumulation in local and systemic infections in control (TRV*‐GUS*) or *NbLDAP1*‐silenced (TRV*‐NbLDAP1*) plants, measured by qRT‐PCR using a primer pair targeting the *CP* gene. The accumulation of viral CP RNA is normalized to the *EF1a* reference gene. For (b, c), data from three experimental replicates were pooled, corresponding to 20–25 independent biological replicates. Box plots display the median (thick line), interquartile range (IQR, box), and whiskers extending to 1.5 × IQR from Q1 (25^th^ percentile) and Q3 (75^th^ percentile). Points indicate the value of each replicate. Points beyond the whiskers are considered as outliers. Asterisks indicate a statistically significant difference according to Wilcoxon rank–sum test (Mann–Whitney *U*). **, *P* < 0.01; ***, *P* < 0.001; ns, not significant when *P* > 0.05.

LDAPs represent a major family of LD surface proteins widely distributed in nonseed tissues of vascular and nonvascular plants. In particular, AtLDAP1 is one of the predominant proteins in Arabidopsis leaf LDs and is well known to be involved in LD biogenesis and regulation of LD size (Gidda *et al*., 2016; Kim *et al*., 2016; Brocard *et al*., 2017; Coulon *et al*., 2020). We thus wondered whether knocking down *LDAP1* expression could affect TuMV infection in *N. benthamiana*. For this purpose, we employed VIGS to silence *NbLDAP1*, as displayed in Fig. S3. VIGS of *NbLDAP1* did not induce any obvious developmental abnormalities (Fig. 4a), but knocked down *NbLDAP1* expression by up to 80% (Fig. 4b). We observed no LD accumulation in leaves of both *NbLDAP1*‐silenced and control (tobacco rattle virus (TRV)‐GUS) plants (Fig. S3c), ruling out the possibility of LD accumulation in response to TRV infection. TuMV‐GFP accumulation was quantified in inoculated leaves by quantitative qRT‐PCR using a TuMV‐capsid (CP) primer pair, which showed that the virus amounts were consistently reduced by half in *NbLDAP1*‐silenced plants compared to control TRV‐GUS plants (Fig. 4c). This suggests a pro‐viral role for NbLDAP1. However, the absence of this trend in systemic‐infected leaves (Fig. 4c) suggests that LDAP1 is involved in the early stages of TuMV infection in *N. benthamiana*.

### TuMV induces LDAP1 protein accumulation and LD accumulation near VRCs in *A. thaliana*

Based on the results presented thus far with *N. benthamiana*, we focused next on the model plant *A. thaliana*, in order to observe the effect, if any, of employing a more stringent repression of *LDAP* expression in another plant host.

**Fig. 5:**
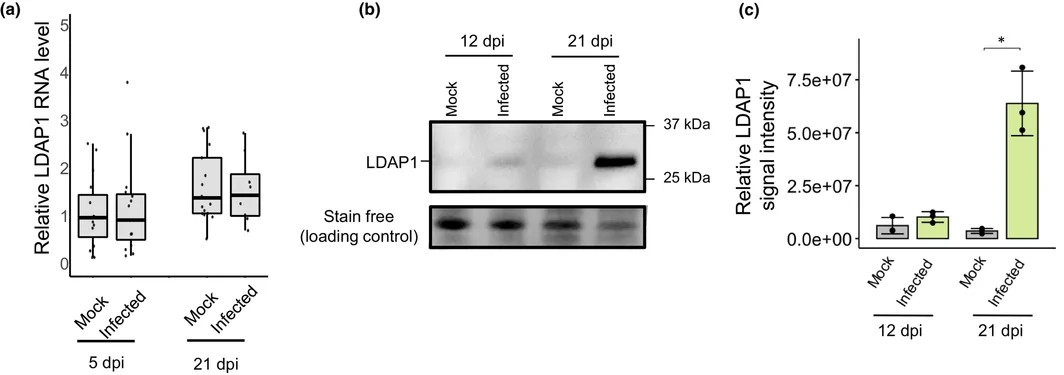
AtLDAP1 proteins but not transcripts accumulate during turnip mosaic virus (TuMV) infection. (a) *AtLDAP1* transcript levels were quantified by quantitative real‐time polymerase chain reaction and normalized to the expression of the *Ubiquitin* housekeeping gene (2^‐Δ(CtLDAP1‐CtUb^) in *Arabidopsis thaliana* leaves inoculated with TuMV‐green fluorescent protein (GFP)//mCherry or mock‐inoculated, at 5 d post‐inoculated (dpi) leaves and 21 dpi (systemically infected leaves). For each time point, gene expression was quantified using a primer pair targeting the *AtLDAP1* gene in leaves sampled from 15 plants, and three independent experiments were performed. Box plots display the median (thick line), interquartile range (IQR, box), and whiskers extending to 1.5 × IQR from Q1 (25^th^ percentile) and Q3 (75^th^ percentile). Points indicate the value of each replicate. Points beyond the whiskers are considered as outliers. Statistical analysis was carried out using R software (Kruskal–Wallis test, not significant). (b) Detection of LDAP1 protein in inoculated (12 dpi) and systemic (21 dpi) leaves of Arabidopsis plants mock‐ or TuMV‐GFP/mCherry–inoculated by immunoblot. Total leaf proteins were extracted and analyzed by immunoblot using anti‐LDAP1 serum. Stain‐free gel images served as loading controls. (c) Relative quantification of AtLDAP1 signal intensity was normalized to total protein signal (stain‐free) using Image Lab software. Data represent three independent biological replicates (means ± SD). Bar plots represent the mean of the measurements; errors bars indicate standard deviation. Points indicate the value of each replicate. Statistical differences were assessed using Dunn's test, *, *P* < 0.05.

We first investigated whether TuMV infection of *A. thaliana* modifies *AtLDAP1* gene expression. No significant change of *AtLDAP1* expression was observed upon TuMV infection using qRT‐PCR (Fig. 5a), similar to *AtLDAP2* and *AtLDAP3* expression (Fig. S4). However, immunoblot analyses of leaf extracts using a serum raised against AtLDAP1 demonstrated that AtLDAP1 protein was more abundant in the infected leaves than in mock controls (Fig. 5b,c), indicating that AtLDAP1 accumulation is induced by TuMV at a posttranscriptional level in Arabidopsis.

**Fig. 6:**
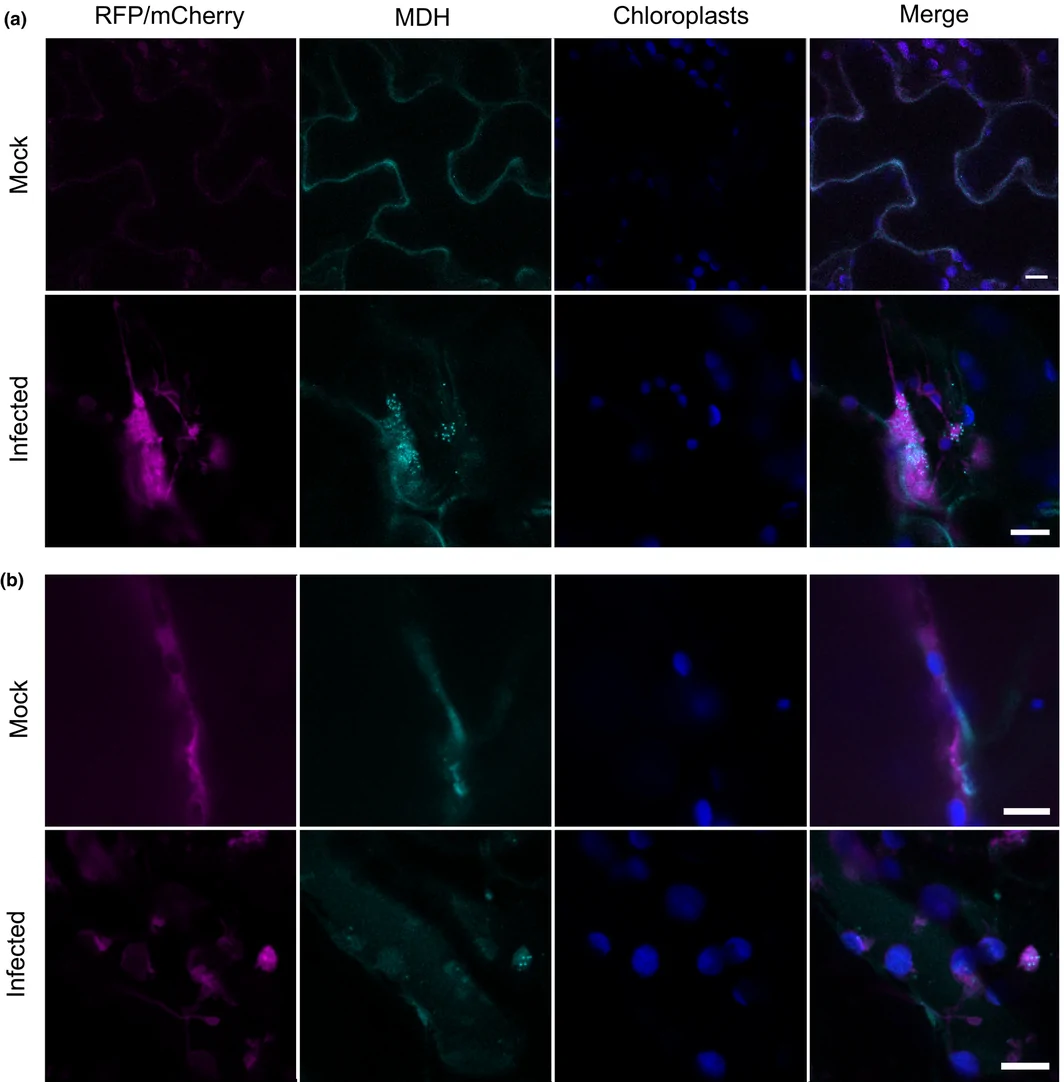
Lipid droplets (LDs) accumulate in close proximity to viral replication compartments (VRCs) in *Arabidopsis thaliana* turnip mosaic virus (TuMV)‐infected leaves. *Arabidopsis thaliana* epidermal cells expressing free red fluorescent protein (RFP) (mock) or inoculated with TuMV/6K2:mCherry construct (infected) were imaged at 6 d post‐inoculation by confocal microscopy after staining with the LD‐specific dye monodensylpentane (MDH). Fluorescence of RFP or 6K2:mCherry (magenta), LDs (cyan), and autofluorescence of Chl (dark blue) are visualized. Numerous LDs are observed in close proximity to 6K2:mCherry‐labeled structures. Bars, 10 μm. (a, b) are representative of observations done in the center of the infection foci (first infected cells) and in the infection foci border (more recently infected cells), respectively. Images are representative of three independent experiments and at least three plants per experiment.

We then employed confocal microscopy to analyze whether TuMV infection results in any alterations in LD biogenesis in *A. thaliana*. As shown in Fig. 6, the induced LDs were located in close proximity to VRCs, as observed in *N. benthamiana* (Fig. 2a), reinforcing our conclusion that LDs are recruited to viral replication sites in TuMV‐infected cells.

### TuMV local propagation is hampered in Arabidopsis *ldap* mutants

**Fig. 7:**
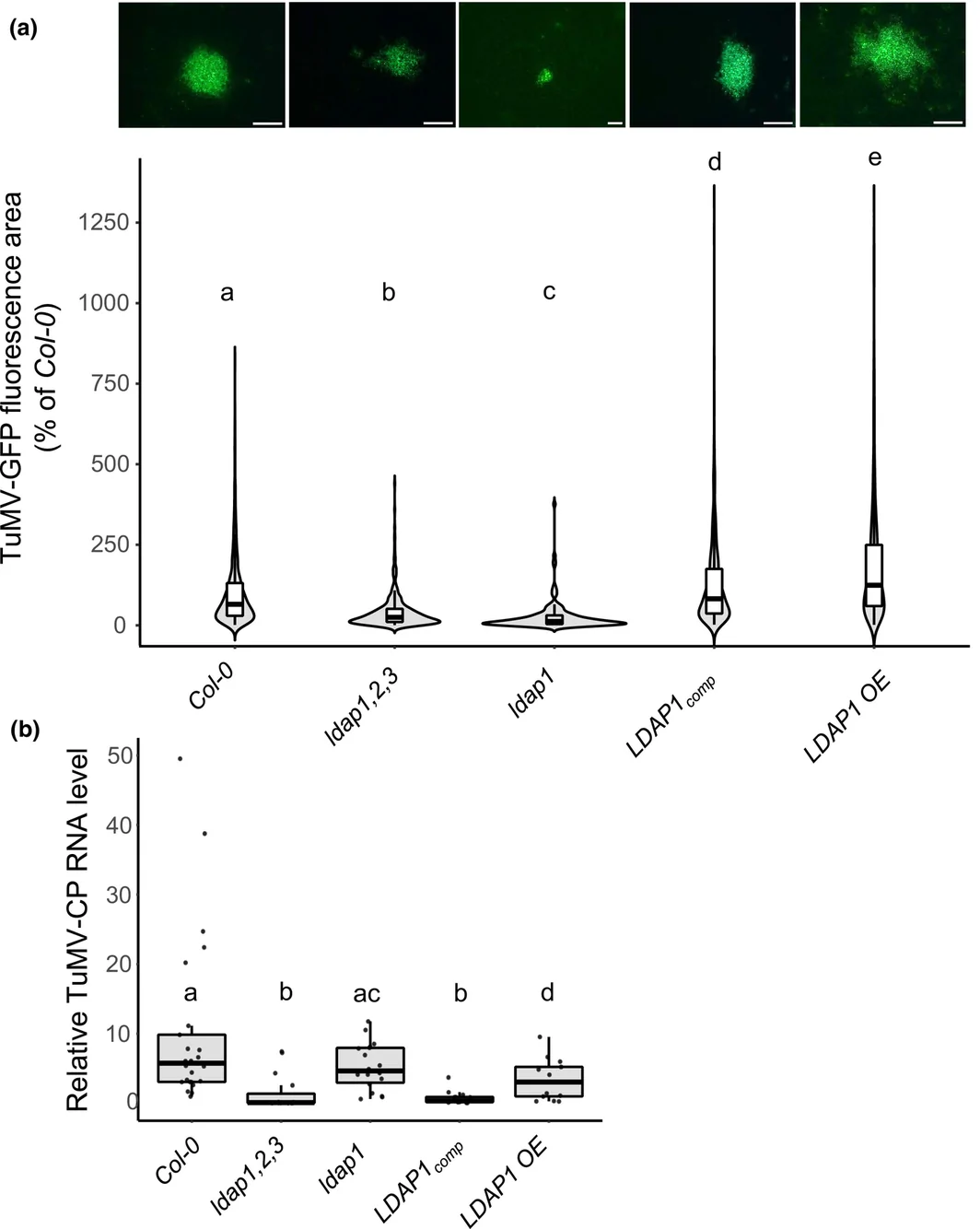
Turnip mosaic virus (TuMV) propagation is reduced in the inoculated leaves of Arabidopsis *ldap* mutants. (a) TuMV propagation was estimated by measuring the area of the infection foci. TuMV‐green fluorescent protein (GFP)//mCherry was inoculated in Arabidopsis leaves of Col‐0, *ldap1*, *ldap1 ldap2 ldap3* (*ldap1,2,3*), *LDAP1*_*pro*_ *:LDAP1* (*LDAP1comp*), and *LDAP1 OE* lines and infection foci, visualized by the GFP fluorescence, were imaged at 5 d post‐inoculation (dpi) in at least three independent experiments. The area of the infection foci was normalized and expressed as a percent of TuMV‐GFP//mCherry propagation area in Col‐0 plants. Different letters indicate statistically different groups with *P* < 0.001; *n* = 1485, 139, 455, 1152, and 1359 for Col‐0, *ldap1*, *ldap1 ldap2 ldap3, LDAP1comp*, and *LDAP1 OE*, respectively. Bars, 300 μm. Violin plots display the distribution of the data using a mirrored density plot, with a central box plot indicating the median and interquartile range (IQR). The width of the violin at each value reflects the data density.(b) TuMV RNA accumulation was quantified by qRT‐PCR at 5 dpi, using a primer pair targeting the CP gene, and expressed relatively to the number of infection foci ratio (2^−Δ(CtCP–CtUb)^/2^−Δ(CtmCherry–CtUb)^). Three technical quantitative real‐time polymerase chain reaction replicates were performed for each sample (*n* = 13–15 per experimental group, three independent experiments). Box plots display the median (thick line), interquartile range (IQR, box), and whiskers extending to 1.5 × IQR from Q1 (25^th^ percentile) and Q3 (75^th^ percentile). Points indicate the value of each replicate. Points beyond the whiskers are considered as outliers.Different letters indicate statistically different groups (*P* < 0.05). Statistical analyses were performed using a Kruskal–Wallis test followed by Dunn's test in R software.

To further explore whether this LD accumulation is a more general plant defense response to viral infection and/or is exploited by the virus for its own benefit, we characterized TuMV propagation in Arabidopsis lines perturbed in LD biogenesis. Toward that end, we focused on LDAP1 by using previously described transgenic lines that are either over‐expressing a mCherry‐tagged version of LDAP1 under the 35S promoter in the WT (Col‐0) background (*LDAP1 OE*) or disrupted in *LDAP1* expression due to a T‐DNA knock‐out insertion in the LDAP1‐coding sequence (*ldap1*) (Gidda *et al*., 2016; Brocard *et al*., 2017). In addition, we generated a triple *atldap* knock‐out mutant line (*ldap1 ldap2 ldap3*) in the *ldap1* background using CRISPR‐/Cas9‐based genome editing, and a complemented *ldap1* mutant line (*LDAP1*_*pro*_ *:LDAP1*), whereby LDAP1‐mCherry driven by the *LDAP1* promoter was introduced into the *ldap1* mutant background (Fig. S1). *LDAP1 OE* plants displayed an increased LD abundance in leaves, whereas the LD number in the single and triple *ldap* mutants was decreased compared to WT, under culture conditions inducing LD biogenesis, such as nitrogen starvation (Gidda *et al*., 2016; Brocard *et al*., 2017; Fig. S5). Each of the abovementioned lines were also inoculated with the TuMV‐GFP//mCherry construct that contains two gene expression cassettes within the T‐DNA: one encoding ER‐localized mCherry (mCherry‐HDEL) and the other a GFP‐tagged TuMV genome. After agroinfiltration into *N. benthamiana* leaves, primary infected cells express both mCherry and GFP, and thereby yield red and green fluorescence. Secondary‐infected cells, infected through viral propagation, display only green fluorescence since only the recombinant TuMV genome carries GFP (Dai *et al*., 2020). To monitor the propagation of the virus (resulting from replication and cell‐to‐cell movement), the area of the GFP‐labeled infection foci was measured in the inoculated leaves at 5 dpi: it was significantly decreased in both *ldap1* and *ldap1 ldap2 ldap3* mutant lines compared to Col‐0, whereas it was significantly increased in the *LDAP1 OE* line (Fig. 7a). TuMV propagation was also restored and even increased in the complemented *LDAP1*_*pro*_ *:LDAP1* line, confirming that the decreased TuMV propagation is due to the mutation of *LDAP1*.

In order to examine specifically the TuMV replication efficiency in the different LDAP mutant lines, the level of accumulation of TuMV genomic RNA was determined by qRT‐PCR using primers specific for the *CP* gene (Table S1) and normalized with the reference gene *mCherry‐HDEL*, as described in Xue *et al*. (2024) and Wu *et al*. (2020). For this purpose, a primer pair specific to the *mCherry‐HDEL* sequence included in the TuMV‐GFP//mCherry construct was used (Table S1), and we confirmed (via RT‐PCR) that these primers did not amplify the *mCherry* sequence fused to *LDAP1* in *LDAP1 OE* and *LDAP1*_*pro*_ *:LDAP1* lines (Fig. S6). By monitoring the CP/mCherry ratio, we could quantify the viral load (accumulation of TuMV genomic RNA) relative to the number of inoculation foci. No significant difference of TuMV RNA accumulation was observed in the *ldap1* mutant compared to Col‐0 (Fig. 7b), suggesting that the decrease in the infection foci surface could be linked to a decrease in movement rather than replication in this mutant. However, a significant decrease of TuMV accumulation was observed in the *ldap1 ldap2 ldap3* mutant (Fig. 7b), indicating that the reduced propagation of TuMV in this line could be due to alteration of both replication and movement.

To specifically evaluate the viral replication efficiency independently of the viral movement, as described in Rocher *et al*. (2022) and Xue *et al*. (2024), we conducted a parallel experiment using the TuMV^W15A^/6K2:mCherry//GFP virus, a mutant which is able to replicate but not move cell‐to‐cell. Thus, TuMV^WT^/6K2:mCherry//GFP served as a nonmutated version of the same viral construct. The accumulation of genomic RNA was similar in Col‐0 and *ldap1* mutant both with TuMV^W15A^/6K2:mCherry//GFP and TuMV^WT^/6K2:mCherry//GFP, indicating that TuMV replication was not altered in the *ldap1* mutant (Fig. S7). While not impacted in the *ldap1* mutant, the accumulation of genomic viral RNA was strongly reduced in the *ldap1 ldap2 ldap3* mutant whether the 6K2 was mutated or not in the virus (Fig. S7), suggesting that LDAP2 and LDAP3 could be involved in TuMV replication, or could compensate LDAP1 loss of function for TuMV replication but not for TuMV movement.

Unexpectedly, a decrease in TuMV RNA accumulation was also observed in *LDAP1 OE* and the complemented *LDAP1*_*pro*_ *:LDAP1* lines (Fig. 7b) although the area of the infection foci was significantly increased compared to Col‐0 (Fig. 7a). This result was confirmed using TuMV^W15A^6K2mCherry//GFP virus, which displayed decreased accumulation in both the *LDAP1 OE* and the complemented *LDAP1*_*pro*_ *:LDAP1* lines (Fig. S7). These results suggest that the increased propagation observed at 5 dpi in the inoculated leaves was probably due to an increase in viral movement rather than an increase in viral replication.

### Arabidopsis LDAPs are involved in TuMV systemic propagation

**Fig. 8:**
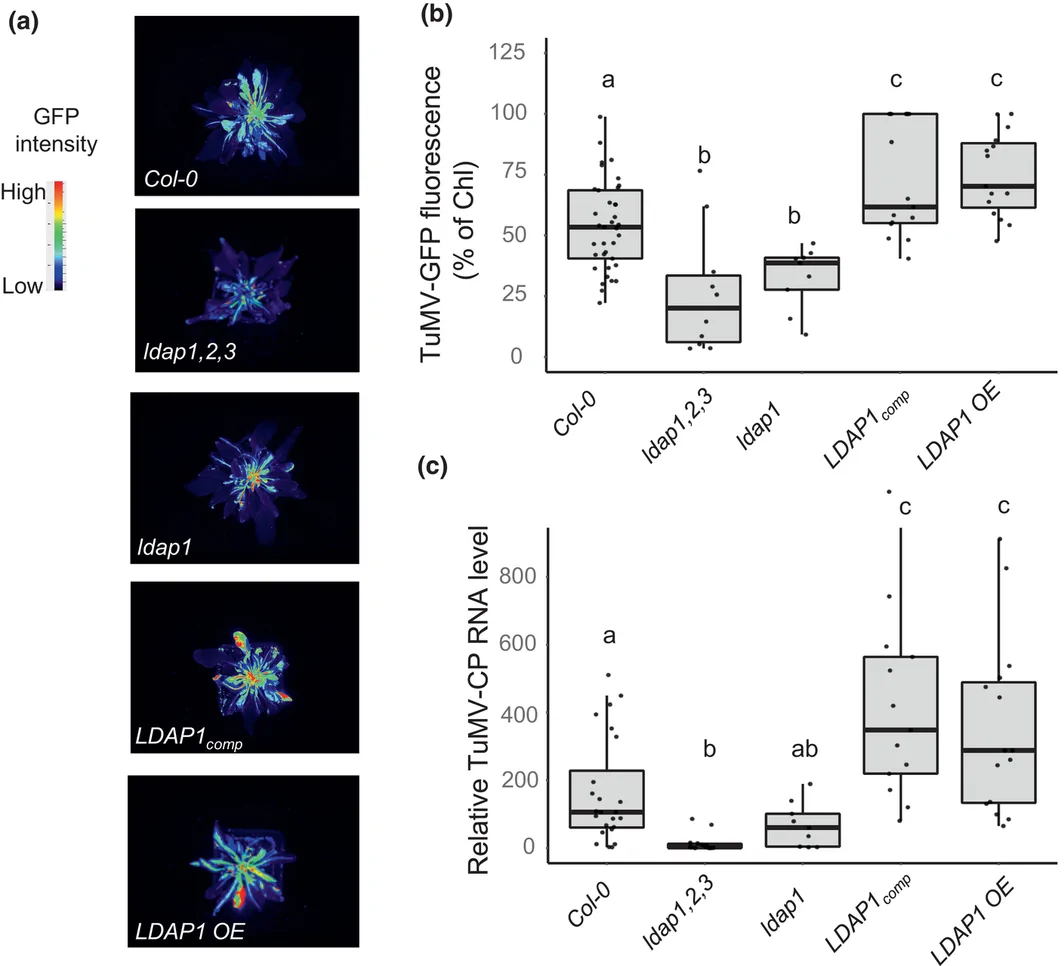
Turnip mosaic virus (TuMV) propagation is reduced in the systemic leaves of Arabidopsis *ldap* mutants but increased in LDAP1 overexpressing leaves. (a) Observation by macroscopy of TuMV propagation in the different *LDAP* genotypes. Arabidopsis Col‐0, *ldap1*, *ldap1 ldap2 ldap3* (*ldap1,2,3*), *LDAP1*_*pro*_ *:LDAP1* (*LDAP1comp*), and *LDAP1 OE* lines were inoculated with TuMV‐green fluorescent protein (GFP)//mCherry, and the GFP fluorescence monitored at 21 d post‐inoculation (dpi) in the whole plant. GFP fluorescence intensity is represented by false colors ranging from blue (low intensity) to red (high intensity). (b) Estimation of TuMV propagation by measuring TuMV‐GFP fluorescence area. The size of the GFP‐fluorescent area is expressed relatively to the total foliar area (estimated by the Chl signal) of five plants per genotype. (c) TuMV RNA accumulation was quantified at 21 dpi by quantitative real‐time polymerase chain reaction, using a primer pair targeting the *CP* gene, and expressed relatively to the *Ubiquitin* reference gene (2^−Δ(CtCP–CtUb)^); three technical qRT‐PCR replicates per sample, *n* = 15 samples by genotype). Box plots display the median (thick line), interquartile range (IQR, box), and whiskers extending to 1.5 × IQR from Q1 (25^th^ percentile) and Q3 (75^th^ percentile). Points indicate the value of each replicate. Points beyond the whiskers are considered as outliers.Different letters indicate significantly different groups identified by Kruskal–Wallis tests followed by Dunn's test in R software (*P* < 0.05).

To determine whether LD biogenesis is necessary for TuMV to move through the vascular system and reach distant tissues in infected plants, we measured viral RNA accumulation and fluorescence area in systemically infected leaves of various *LDAP* mutants. To do so, we used the GFP signal as a marker for viral propagation and performed fluorometric analysis (Fig. 8a) as described by Zafirov *et al*. (2021). TuMV propagation in the systemically infected leaves at 21 dpi was slightly but significantly delayed in *ldap1* and *ldap1 ldap2 ldap3* mutants compared to Col‐0 (Fig. 8b), while virus propagation was accelerated in the overexpressing and complemented lines. These results are in agreement with the measurements of the area of infection foci at 5 dpi (Fig. 7a) and suggest that altering the cell‐to‐cell movement of TuMV in the inoculated leaves alters the systemic spread of the virus. No significant decrease of TuMV RNA accumulation was observed in *ldap1* compared to Col‐0 in the systemic leaves (Fig. 8c), but a significant decrease was observed in *ldap1 ldap2 ldap3* where the systemic propagation was severely reduced compared to Col‐0. On the other hand, the accumulation of TuMV in *LDAP1 OE* and *LDAP1*_*pro*_ *:LDAP1* lines was increased compared to Col‐0, suggesting the involvement of LDAPs in TuMV systemic propagation.

### TuMV propagation is impaired in other Arabidopsis lines altered in LD biogenesis: the *seipin* mutants

We investigated next whether perturbing LD biogenesis by disrupting *SEIPIN* gene expression also had an impact on TuMV propagation using the *seipin2 seipin3* mutant, which displays fewer but enlarged LDs in seedlings (Taurino *et al*., 2018) and leaves (Fig. S5), and a SEIPIN1 OE line which displays an increase in LD abundance (Cai *et al*., 2015).

**Fig. 9:**
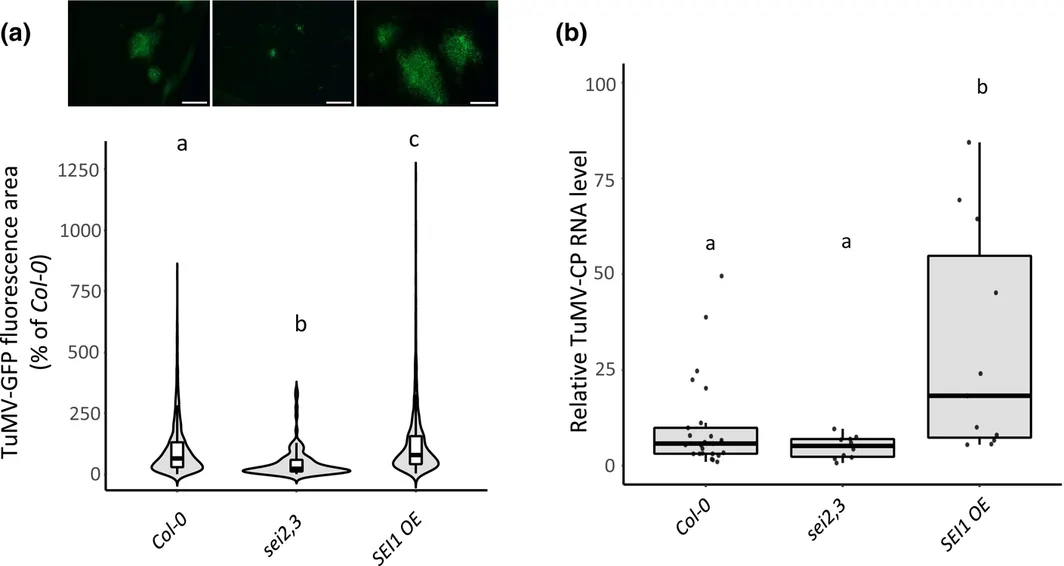
Turnip mosaic virus (TuMV) propagation is reduced in the inoculated leaves of Arabidopsis *seipin* mutants but increased in the *SEIPIN* overexpressing line. (a)TuMV propagation was estimated by measuring the area of the infection foci. TuMV‐green fluorescent protein (GFP)//mCherry was inoculated in Arabidopsis leaves of Col‐0, *seipin2 seipin3* (*sei2,3*), and *SEIPIN1 OE* (*SEI1 OE*) lines and infection foci were imaged at 5 d post‐inoculation in at least three independent experiments. The size of the infection foci was normalized and expressed as a percentage of theTuMV‐GFP//mCherry infection foci area in Col‐0 plants. Violin plots display the distribution of the data using a mirrored density plot, with a central box plot indicating the median and interquartile range (IQR). The width of the violin at each value reflects the data density. Different letters indicate statistically different groups (*P* < 0.001); *n* = 716, 109, and 1006 for Col0, *seipin2 seipin3*, and *SEIPIN1 OE* lines, respectively. Bars, 300 μm. (b) TuMV RNA accumulation was determined by quantitative real‐time polymerase chain reaction, using a primer pair targeting the *CP* gene, and expressed relatively to the number of infection foci (2^−Δ(CtCP–CtUb)^/2^−Δ(CtmCherry–CtUb)^). Three technical replicates were performed for each sample (*n* = 13–15 per experimental group, three independent experiments). Box plots display the median (thick line), interquartile range (IQR, box), and whiskers extending to 1.5 × IQR from Q1 (25^th^ percentile) and Q3 (75^th^ percentile). Points indicate the value of each replicate. Points beyond the whiskers are considered as outliers.Different letters indicate statistically different groups (*P* < 0.05). Statistical analyses were performed using a Kruskal–Wallis test followed by Dunn's test in R software.

Compared to Col‐0, the area of the infection foci induced by TuMV‐GFP//mCherry was significantly decreased in the *seipin2 seipin3* mutant, whereas it was significantly increased in *SEIPIN1 OE* line (Fig. 9a). However, TuMV accumulation was similar in the *seipin2 seipin3* mutant and Col‐0 (Fig. 9b). Similarly, the accumulation of the nonmoving TuMV^W15A^/6K2:mCherry//GFP virus was not impacted by the inactivation of *SEIPIN2* and *SEIPIN3* genes (Fig. S7). These results suggest that the decreased spread of TuMV in *seipin2 seipin3* mutant is not linked to a reduced level of viral genomic accumulation (Fig. 9b). On the contrary, *SEIPIN1* overexpression favored both TuMV propagation and viral accumulation in the inoculated leaves (Fig. 9a,b) but did not impact the replication of TuMV^W15A^/6K2:mCherry//GFP (Fig. S7). These results suggest that the increase in viral accumulation of TuMV in *SEIPIN1 OE* is linked to an increased cell‐to‐cell movement of the virus, comparable to what we observed in the *LDAP1 OE* mutant.

**Fig. 10:**
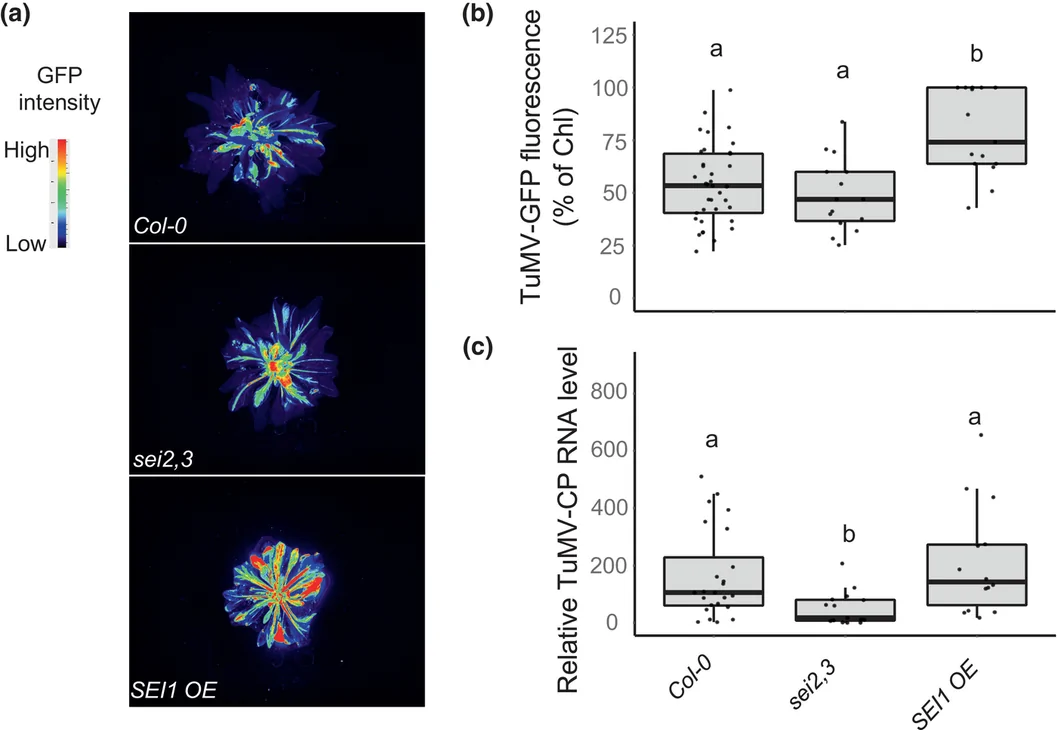
Turnip mosaic virus (TuMV) propagation is reduced in the systemic leaves of Arabidopsis *seipin* mutants but increased in the *SEIPIN* overexpressing line. (a) Observation of TuMV propagation in the different *SEIPIN* genotypes by macroscopy. Arabidopsis Col‐0, *seipin2 seipin3 (sei2,3)*, and *SEIPIN1 OE* (*SEI1 OE*) lines were inoculated with TuMV‐green fluorescent protein (GFP)//mCherry, and the GFP fluorescence was monitored at 21 d post‐inoculation (dpi) in the whole plant. GFP fluorescence intensity is represented by false colors ranging from blue (low intensity) to red (high intensity). (b) Estimation of TuMV propagation by measuring TuMV‐GFP fluorescence area. The size of the GFP‐fluorescent area is expressed relatively to the total foliar area (estimated by the Chl signal) of five plants per genotype. (c) TuMV RNA accumulation was quantified at 21 dpi by quantitative real‐time polymerase chain reaction, using a primer pair targeting the *CP* gene, and expressed relatively to the *Ubiquitin* reference gene (2^−Δ(CtCP–CtUb)^; 3 technical replicates per sample, *n* = 15 samples by genotype). Box plots display the median (thick line), interquartile range (IQR, box), and whiskers extending to 1.5 × IQR from Q1 (25^th^ percentile) and Q3 (75^th^ percentile). Points indicate the value of each replicate. Points beyond the whiskers are considered as outliers. Different letters indicate significantly different groups (*P* < 0.05). Statistical analyses were performed using a Kruskal–Wallis test followed by Dunn's test in R software.

The systemic spreading of TuMV was accelerated in the *SEIPIN1 OE* line, in agreement with a pro‐viral role of SEIPIN1 in TuMV movement (Fig. 10a,b). Although the fluorometric analysis of the systemic spread of TuMV in *seipin2 seipin3* did not show any obvious difference in comparison to Col‐0, we observed a lower accumulation of TuMV in the systemically infected leaves (Fig. 10c), which could indicate delayed movement of the virus. Taken together, these results showed that similar to LDAPs, SEIPINs have a positive effect on TuMV movement in Arabidopsis.

## Discussion

### Impact of viral infection on leaf lipid metabolism

In this study, we provided compelling evidence that LDs are not only responsive to infection by TuMV but also may facilitate the virus life cycle in plants. For instance, we observed the accumulation of NLs together with an increased LD abundance in *N. benthamiana* upon TuMV infection, which suggests that TuMV can disrupt normal lipid metabolism and storage by exploiting LDs. Consistent with this premise, membrane remodeling is well known to be a key feature of viral infection in both animal and plant cells, and the lipid composition of plant virus replication sites is known to be highly regulated (Fernández de Castro *et al*., 2016; Jin *et al*., 2018). The requirement for unsaturated fatty acids (FAs) in replication vesicle formation, for example, has been well demonstrated in brome mosaic virus‐infected cells (Lee *et al*., 2001), and tombusviruses were shown to enrich their VRCs in sterols and phosphatidylethanolamine (Barajas *et al*., 2014). The only study suggesting that a plant virus could exploit LDs to regulate FA metabolism involved RBSDV, which is a multipartite double‐stranded RNA virus (Wang *et al*., 2024). This virus induces elevated amounts of polyunsaturated FAs in maize, as also occurs in plants in response to other biotic stresses (Kachroo & Kachroo, 2009; Lim *et al*., 2017). An interaction between the RBSDV‐core capsid protein P8 with a maize LDAP, ZmLDAP2, is proposed to prevent the degradation of ZmLDAP2 by the proteasome. ZmLDAP2 stabilization is then considered to help maintain high levels of polyunsaturated FAs, which are critical for RBSDV replication and systemic spread of the virus (Wang *et al*., 2024). Whether RBSDV infection enhances the biogenesis of LDs in maize still remains to be determined. Nonetheless, we showed that during TuMV infection in *N. benthamiana*, the overall C18:3 content in total lipids–which include both neutral and polar lipids – remains unchanged compared to mock‐treated plants (Fig. S8). This contrasts with RBSDV infection, where C18:3 content is 3.5‐fold higher in infected leaves than in mock conditions (Wang *et al*., 2024). However, TuMV infection specifically induces an increase of the C18:3 content in the NL fraction (Fig. 1b). As *c*. 75% of C18:3 present in polar lipids come from galactolipids in thylakoids (Li‐Beisson *et al*., 2013), it is possible that the FAs used for TAG synthesis in response to TuMV infection are mostly derived from galactolipids, as described in response to nitrogen starvation (Coulon *et al*., 2024).

### Role of LDs in the assembly and/or function of VRCs during viral infection

It has been well documented that mammalian viruses from different families, such as HCV, Dengue virus, SARS‐CoV‐2, or poliovirus, extensively manipulate LDs for viral replication and virion production (Lee *et al.*, 2019; Laufman *et al*., 2019; Cloherty *et al*., 2020; Cesar‐Silva *et al*., 2023; Hsia *et al*., 2024). Our observations clearly show that in TuMV‐infected plants, there is a conspicuous association of newly formed LDs with VRCs (Figs 2 and 3). This proximity of LDs to TuMV VRCs suggests that LDs serve to support the assembly and function of VRCs. Additional evidence suggesting the involvement of LDs in VRC organization comes from Wan *et al*. (2015), whereby so‐called ‘electron‐dense bodies’ were detected in TuMV‐infected cells, closely associated with viral particle assembly. Given these results and the new findings from this study highlighting the behavior of LDs in plant–virus interactions, it is plausible that TuMV exploits LDs for virion assembly, akin to the strategies that are utilized by other more well studied mammalian viruses. That is, LDs could facilitate the formation of TuMV‐VRC, either by providing the necessary local lipid supply for vesicle formation and/or influencing membrane curvature through the recruitment of key lipids and/or structural proteins. In support of this premise, recent LD proteomics studies of LD‐enriched fractions from plants (Veerabagu *et al*., 2021; Omata *et al*., 2024) revealed the presence of the reticulon protein 3 (RTN3) and the ER fusogen ROOT HAIR DEFECTIVE 3 (RHD3). Interestingly, RHD3 and another reticulon‐like protein, HVA22a, both enhance replication and propagation of TuMV by facilitating the maturation of 6K2‐induced vesicles (Movahed *et al*., 2019; Xue *et al*., 2024). These findings raise the possibility that LDs may serve as scaffolds during early steps of TuMV VRC biogenesis, and provide, in addition to lipids, proteins, such as membrane curving reticulons, that are necessary for their assembly.

### LDAPs and SEIPINs play an essential role in TuMV propagation

*LDAP* genes are known to be selectively upregulated in response to abiotic stress or during specific developmental stages, such as senescence (Brocard *et al*., 2017). In addition, LDs tend to proliferate in response to abiotic stress, and the ectopic OE of LDAPs increases LD abundance in leaves and enhances drought tolerance (Kim *et al*., 2016; Brocard *et al*., 2017; Laibach *et al*., 2018). By contrast, the loss of function of any *LDAP* gene leads to a reduction in LD numbers in leaves (Gidda *et al*., 2016; Kim *et al*., 2016; Brocard *et al*., 2017). Our study established that TuMV infection does not influence LDAP1 mRNA levels (see Figs 5, S3), unlike during leaf senescence (Brocard *et al*., 2017) or temperature stress (Gidda *et al*., 2016; Kim *et al*., 2016). However, we did show that the amount of LDAP1 protein increases during TuMV infection (Fig. 5b,c). This suggests that, during TuMV infection, *LDAP1* expression is regulated at the post‐transcriptional level and/or LDAP1 degradation is prevented by a yet‐unknown mechanism, similar to what has been described for either the Zika virus, which stabilizes DGAT2 protein (Luo *et al*., 2024), or the RBSDV virus and ZmLDAP2 (Wang *et al*., 2024).

We showed that disruption of only the *LDAP1* gene in Arabidopsis was sufficient to significantly decrease the area of TuMV infection foci (consequence of cell‐to‐cell movement and replication, Fig. 7a) but without perturbing TuMV genomic RNA accumulation (Figs 7b, S7). Conversely, TuMV propagation was favored by *LDAP1* overexpression in Arabidopsis (Fig. 7a). These results suggest that LDAP1 plays a pro‐viral role in TuMV infection, and that the loss‐of‐function of *LDAP1* negatively affects TuMV cell‐to‐cell movement in inoculated leaves but does not impact drastically the level of viral RNA accumulation. Our experiments on virus‐induced *LDAP1* silencing also suggest that *NbLDAP1* contributes to TuMV infection in *N. benthamiana*. Indeed, partial silencing of *NbLDAP1* resulted in a significant reduction of viral RNA accumulation in local infection. However, this was not observed in systemic infection (Fig. 4c). Notably, *NbLDAP1* was knocked down using VIGS, with transcript level reduced *c*. 80%, therefore some NbLDAP1 proteins may still be synthesized, which may continue to support virus infection in *N. benthamiana*. Moreover, VIGS produces a mosaic of silenced and nonsilenced cells within any given infected leaf. Thus, when viral titers are measured at the whole‐tissue level by qRT‐PCR, the presence of unsilenced regions could dilute the apparent effect of *LDAP1* knockdown on virus accumulation in the systemic leaves.

We showed also that by knocking‐out all three of the *LDAP* genes in Arabidopsis, the area of TuMV infection foci was significantly decreased (Fig. 7a) and TuMV genomic RNA accumulation significantly lowered for the three viruses tested (i.e. TuMV‐GFP//mCherry, TuMV^W15A^/6K2:mCherry//GFP and TuMV^WT^/6K2‐mCherry//GFP), compared to Col‐0 (Figs 7b, S7). This suggests that either LDAP2 and/or LDAP3 also play a positive role in viral infection or are sufficient to compensate *LDAP1* loss of function for viral replication but not for movement in Arabidopsis.

Unexpectedly, a decrease in TuMV accumulation in the inoculated leaves was also observed in the *LDAP1 OE* and complemented *LDAP1*_*pro*_ *:LDAP1* lines compared to Col‐0, although the area of the infection foci was significantly increased in both mutants (Fig. 7b). TuMV^W15A^/6K2:mCherry//GFP replication was similarly reduced in the *LDAP1 OE* and *LDAP1pro:LDAP1* lines (Fig. S7). While we do not have any clear explanation for these observations, we can hypothesize that although TuMV genomic RNA accumulation was decreased in these lines, the viral protein levels remained high enough to support efficient cell‐to‐cell movement of the virus. Moreover, as potyviruses (and TuMV) have been shown to hijack the host‐cell translational machinery, we may not expect a strict correlation between virus mRNA and viral protein accumulation levels, and it could be that the amounts of viral RNAs and proteins are uncoupled (Zafirov *et al*., 2023). There are other examples from previous studies on poty‐ and potexviruses in different genetic plant backgrounds, where larger infection foci are not linked to higher viral RNA accumulation (Rocher *et al*., 2022) or smaller infection foci to lower viral RNA accumulation (Raffaele *et al*., 2009; Rocher *et al*., 2022; Xue *et al*., 2024). Indeed, in these studies, the levels of viral accumulation are not modified and therefore the movement of the virus was considered to be altered. In another study on TuMV mutants (Deng *et al*., 2015), one mutant (named CI‐m18) was able to move cell to cell although its replication was strongly decreased, whereas another mutant (named CI‐m40) displayed a higher replication level but did not move cell to cell. These examples illustrate again that a strict (positive) correlation between the area of the infection foci and the level of viral RNA detected by qRT‐PCR in the inoculated leaves is not always observed.

The disruption of viral infection in *ldap* mutant plants could indicate a direct effect of LDAPs on viral spread, through interaction with viral proteins at the LDs, similar to what has been described for some animal viruses (Tang *et al*., 2014; Griseti *et al*., 2024). However, the impact of *LDAP* dysregulation on viral infection could also originate not from direct interaction of LDAPs with a TuMV protein, but from the effect of LDAPs on regulating the size and number of LDs. That is, we frequently observed in *ldap* mutants that LDs have decreased mobility and a reduced surface‐to‐volume ratio (Fig. S5), which potentially limits the availability for LD contacts. Thus, viral infection could be affected in *ldap* mutants not because of the absence of LDAP *per se*, but due to the difference in LD size and number.

Like LDAP1, SEIPINs were also shown to influence TuMV propagation (Fig. 9). The heterologous expression of Arabidopsis SEIPINs in *N. benthamiana* leaves greatly increases the number of LDs, and SEIPIN1 favors the formation of a greater proportion of larger LDs, while SEIPIN2 and SEIPIN3 overexpression produce more normal‐sized or smaller LDs, respectively (Cai *et al*., 2015). Here we showed that *SEIPIN1* overexpression led to enhanced viral propagation, whereas in the *seipin2 seipin3* double mutant there was a conspicuous defect in TuMV infection (Figs 9, 10, S7). This suggests that SEIPINs, like LDAPs, are involved in TuMV infection. Further investigations are needed to determine whether the impact of SEIPINs and LDAPs on TuMV infection results from a direct interaction with viral proteins, is a consequence of dysregulation of LD biogenesis, or both.

### Do LDs play a role in TuMV movement?

Our results suggest that LDs, or at least LD‐associated proteins (i.e. LDAPs and SEIPINs), play a positive role in viral movement. The cell‐to‐cell movement of plant viruses is a key step of the viral infection whereby plasmodesmata (PD), the symplastic tunnels between cells, are the gateway for this movement (Reagan & Burch‐Smith, 2020). For potyviruses and TuMV in particular, replication and cell‐to‐cell movement are tightly coupled (for review Xue *et al*., 2023), and recent proteomic studies have shown that plant LD proteins are involved in PD function and potyvirus movement. Pertinent examples of these proteins include reticulons (RTN3), atlastins (RHD3), syntaxins, synaptotagmins, and cytoskeletal components, many of which are well known to facilitate PD membrane reshaping and/or vesicle docking (Veerabagu *et al*., 2021; Omata *et al*., 2024). In particular, LDs can serve as vehicles for transporting proteins to PD, as well as contribute to the regulation and maintenance of the PD structure (van der Schoot *et al*., 2011; Paul *et al*., 2014). Additional investigation is required to establish whether LDs participate in potyviral movement by transporting viral proteins or viral vesicles to PDs.

### Is there a pro‐viral role for LDs with other plant viruses?

The question of whether LDs play a pro‐viral role with viruses other than TuMV remains open and is under investigation in our laboratories. Interestingly, in a previous paper describing the ultrastructural changes induced by MNSV, which belongs to the genus *Gammacarmovirus*, the authors observed viral altered replication organelles frequently close to LDs (Gómez‐Aix *et al*., 2015), suggesting a possible role of LDs in MNSV replication, assembly, or other processes associated with viral infection. Moreover, in other more recent studies, the fijivirus RBSDV was shown to manipulate LD‐associated proteins in order to promote infection (Wang *et al*., 2024), while the nucleocapsid N protein of the VmNSRV1 virus was demonstrated to target to LDs in both fungal and plant cells (Dai *et al*., 2024). The N protein of this ‘phyto‐mycovirus’ binds to LDs and induces their architectural rearrangement, which might optimize the local lipid environment for viral replication though the mechanisms that are as‐of‐yet unknown.

Collectively, our findings reveal a pro‐viral function of LDs in a plant/(+) RNA virus pathosystem and suggest that the LD biogenetic proteins, LDAP and SEIPIN, are both co‐opted by TuMV to optimize its environment for successful infection, in particular cell‐to‐cell movement. This work not only expands our understanding of plant–virus interactions but also positions LDs as potential targets for antiviral strategies.

## Competing interests

None declared.

## Author contributions

NA, DC, CB, J‐LG and SG‐R conceptualized the study. LJ, NA, MB, LS, SS, MC, NMD, SKG, RTM, SA, J‐LG, DC, CB and SG‐R performed tools and methodology. LJ, NA, MB, SS, DC, CB and SG‐R performed formal analysis. LJ, NA, MB, LS, SS, MC, VS, CQ, TS, DC and CB investigated the study. CB and SG‐R led the writing of the original draft. LJ, NA, MB, SS, DC and CB contributed to figures. NA, J‐LG, CB and SG‐R contributed to the writing of review and editing. NA, SS, DC, CB and SG‐R supervised this study. SG‐R is contributed to project administration. CB, J‐LG and SG‐R contributed to funding acquisition. All authors have read and agreed to the published version of the manuscript. LJ and NA share the first authorship. CB and SG‐R share the last authorship.

## Disclaimer

The New Phytologist Foundation remains neutral with regard to jurisdictional claims in maps and in any institutional affiliations.

## Supporting information


**Fig. S1** Validation of the different Arabidopsis *LDAP* mutant lines.
**Fig. S2** Confocal observations of turnip mosaic virus‐green fluorescent protein‐inoculated *Nicotiana benthamiana* leaves.
**Fig. S3** Experimental design for local and systemic turnip mosaic virus infection assays in *NbLDAP1*‐silenced *Nicotiana benthamiana* plants and LD accumulation upon TRV infection carrying virus‐induced gene silencing target constructs.
**Fig. S4** *AtLDAP2* and *AtLDAP3* expression levels during turnip mosaic virus infection.
**Fig. S5** Confocal observations of *ldap1,2,3* and *sei2,3* mutants after 7 d of nitrogen starvation.
**Fig. S6** Control by real‐time polymerase chain reaction of the specificity of primers designed to amplify the mCherryHDEL viral sequence.
**Fig. S7** Evaluation of the accumulation of turnip mosaic virus genomic RNA levels relative to green fluorescent protein expression by quantitative real‐time polymerase chain reaction.
**Fig. S8** Analysis of C18:3 content in total lipids in turnip mosaic virus‐infected *Nicotiana benthamiana* leaves.

**Table S1** Primers sequences.
**Table S2** Nucleotide identity percentage of LDAP1 regions.
**Table S3** Measurements of the area of the infection foci or green fluorescent protein fluorescence.
**Table S4** Quantitative real‐time polymerase chain reaction data used to estimate turnip mosaic virus RNA accumulation.
**Table S5** Lipid data used to generate Figs 1, S8.
Please note: Wiley is not responsible for the content or functionality of any Supporting Information supplied by the authors. Any queries (other than missing material) should be directed to the *New Phytologist* Central Office.

## Data Availability

The datasets used and/or analyzed during the current study are provided within the main text and in the Supporting Information (Tables S3–S5). The accession numbers for the genes discussed in this article are as follows: AtSEIPIN1 (At5g16460), AtSEIPIN2 (At1g29760), AtSEIPIN3 (At2g34380), AtLDAP1 (At1g67360), AtLDAP2 (At2g47780), and AtLDAP3 (At3g05500).
