# Supplementary material for: Turnip mosaic virus utilizes the lipid droplet biogenesis machinery to facilitate its propagation in plants
Source: New Phytol. 2026 Jun 9;252(2):797–816. doi: 10.1111/nph.71321 (PMC13583387; doi:10.1111/nph.71321)
Supplement: Supplementary file 1 [file NPH-252-797-s002.pdf]

New Phytologist Supporting Information

Article title:

Turnip mosaic virus utilizes the lipid droplet biogenesis machinery to facilitate its propagation in plants

Authors: Léna Jambou[$], Nathalie Arvy[$], Marguerite Batsale, Luc Sofer, Sara Shakir, Marielle Cochet, Vincent Simon, Charlotte Quinteau, Thomas Scotti, Nathan M. Doner, Satinder K. Gidda, Robert T. Mullen, Sabine D'Andréa, Jean-Luc Gallois, Denis Coulon[*], Claire Bréhélin[*] and Sylvie German-Retana[*]

Article acceptance date: 10 May 2026

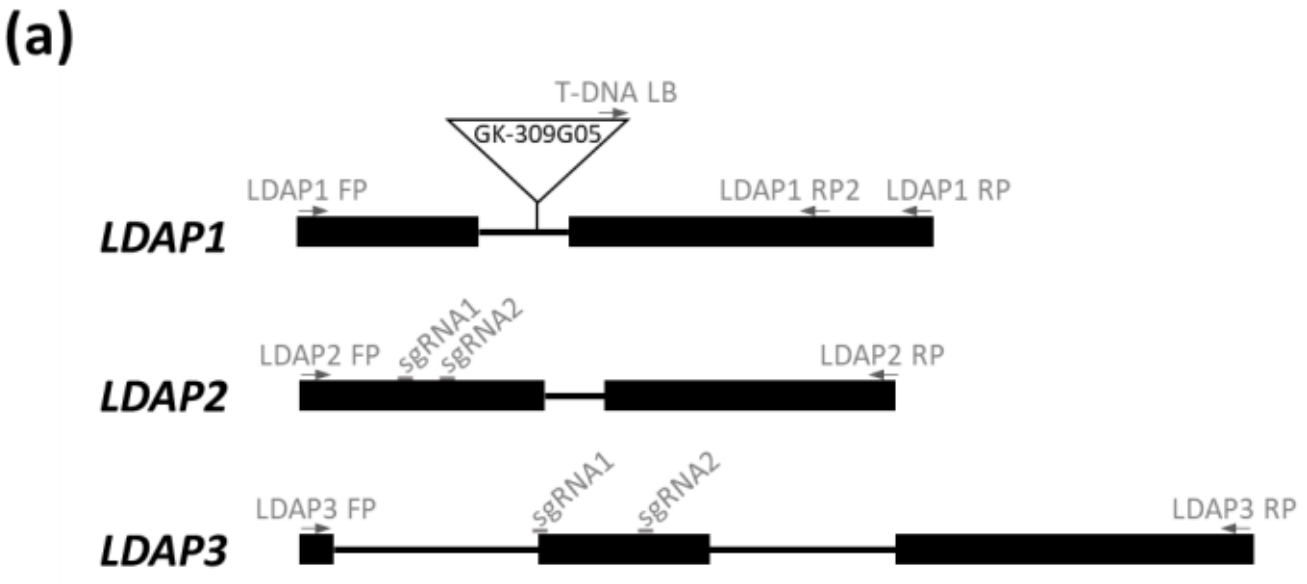

Fig. S1 **Characterization of Arabidopsis *LDAP* lines** (see next page for full legend).

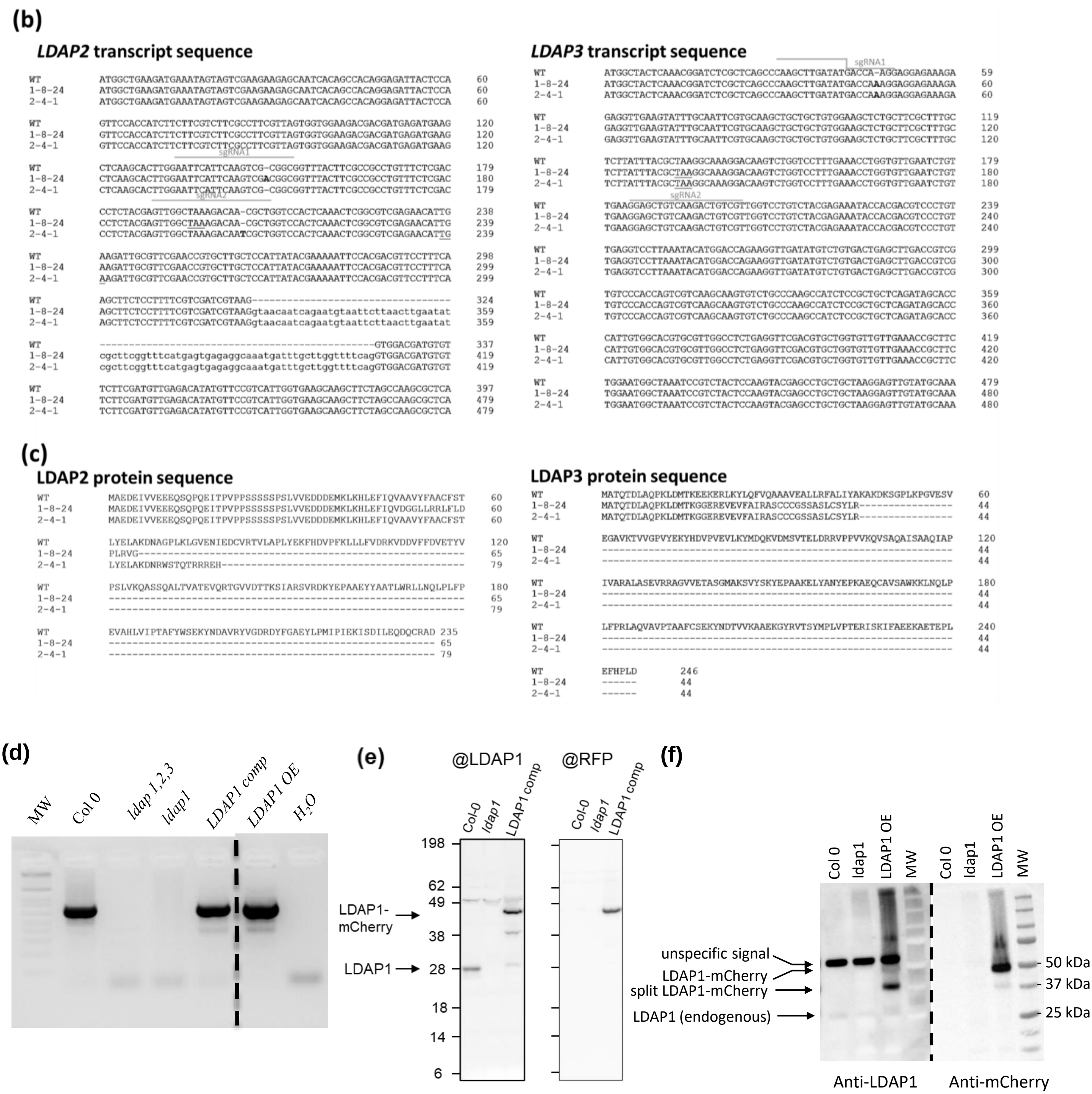

Fig. S1 Characterization of Arabidopsis *LDAP* lines. (**a**) Illustrations of the Arabidopsis *LDAP1, LDAP2, and LDAP3* genes depicting the relative positions of the introns (black lines) and exons (black boxes) in each open reading frame. Shown also are the relative locations of the *ldap1* T-DNA insert (GABI-Kat line GK-309G05) and the sgRNA target sequences in *LDAP2* and *LDAP3*. **(b)** Sequence comparisons of the *LDAP2* and *LDAP3* transcripts (based on sequencing of RT-PCR products) in WT and two independent *ldap* triple mutant lines, named 1-8-24 and 2-4-1; note that all experiments described in this study were performed with the 1-8-24 mutant line. Single-base insertions in both genes are indicated in bold and the resulting premature stop codons in each transcript are underlined. sgRNA sequences are denoted by a gray bar above the WT sequences. Note that the LDAP3 sgRNA1 spans an intron-exon boundary (refer to [a]). Also shown in lower case is the sequence of the retained intron in the *LDAP2* transcript in both mutant lines. **(c)** Deduced amino acid sequences of WT and mutant LDAP2 and LDAP3 proteins. The frameshift mutations eventually result in premature stop codons (refer to [b]) and, consequently, modified versions of the LDAP2 and LDAP3 proteins in both mutant lines. **(d)** The expression of *LDAP1* gene in the different *ldap1* mutants were controlled by RT-PCR using primers to amplify the *LDAP1* cDNA sequence. **(e)** Validation of the *LDAP1* complemented line by immunoblot. Total protein extracts from four-day-old plantlets were analyzed by immunoblot using anti-LDAP1 and anti-RFP antibodies. Equivalent amounts, based on the number of plantlets, were loaded per lane. **(f)** Detection of LDAP1-mCherry protein in *LDAP1 OE* leaves by immunoblot. 50 µg of total protein extracts from Col-0, *ldap1* and *LDAP1OE* leaves were analyzed by immunoblot using anti-LDAP1 serum. The fusion protein LDAP1-mCherry with an expected size of 52 kDa was hardly visible below an unspecific signal, but a degradation product of 37 kDa was detected in the *LDAP1 OE* leaves. The presence of the mCherry tag in the full and split forms was confirmed with a commercial antibody raised against mCherry.

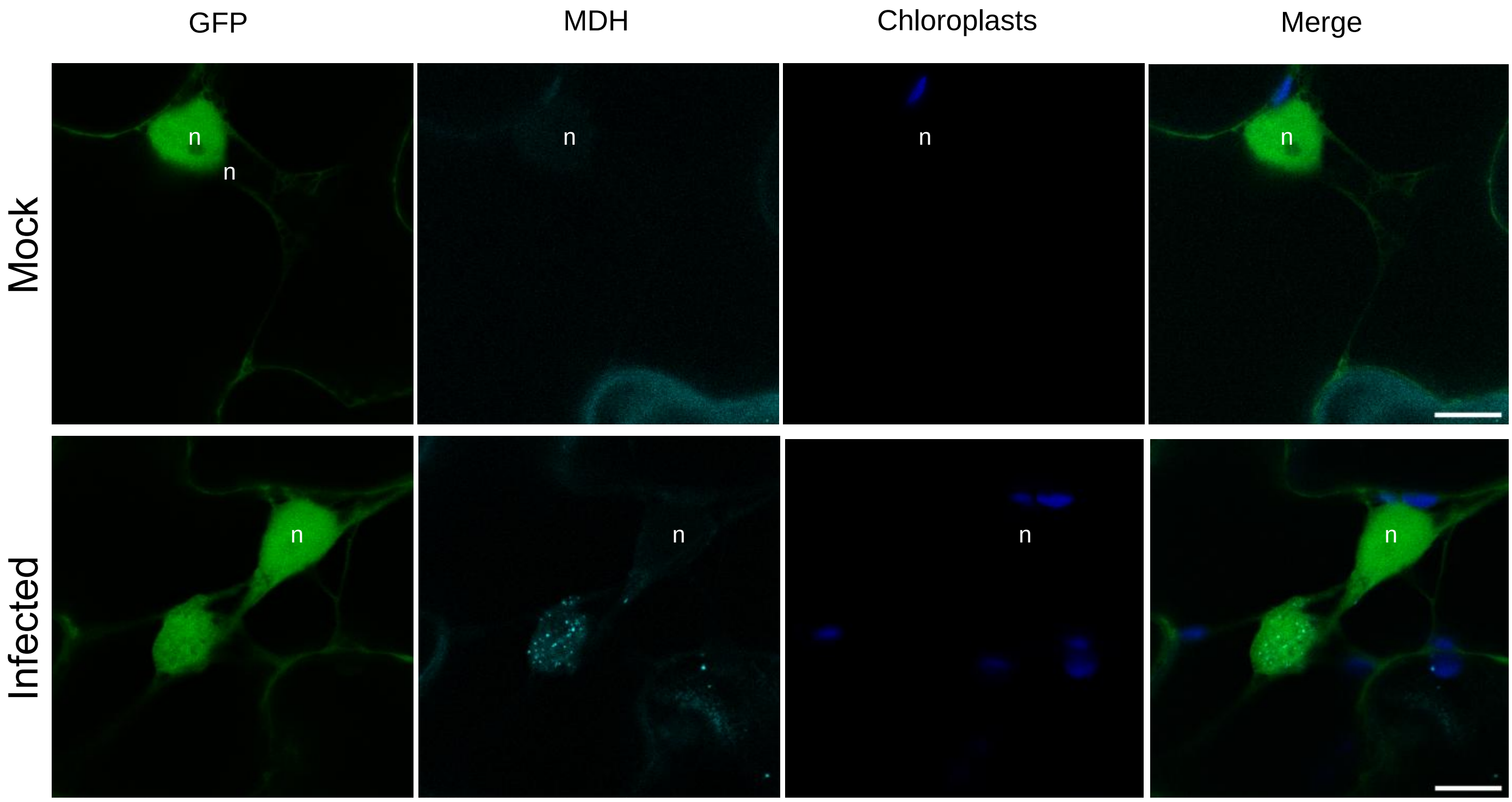

<u>Fig. S2</u> LDs are accumulated during TuMV-GFP infection in *N. benthamiana* leaves*.* Mock- and TuMV-GFP–inoculated *N. benthamiana* leaves were observed at 3 dpi by confocal microscopy. The green channel shows free GFP expressed under mock conditions and GFP expressed during TuMV-GFP infection. Subsequent panels display LDs stained with MDH (cyan), chlorophyll autofluorescence (dark blue), and the merged fluorescence image. The images shown are representative of observations performed on three independent biological replicates. n = nucleus.

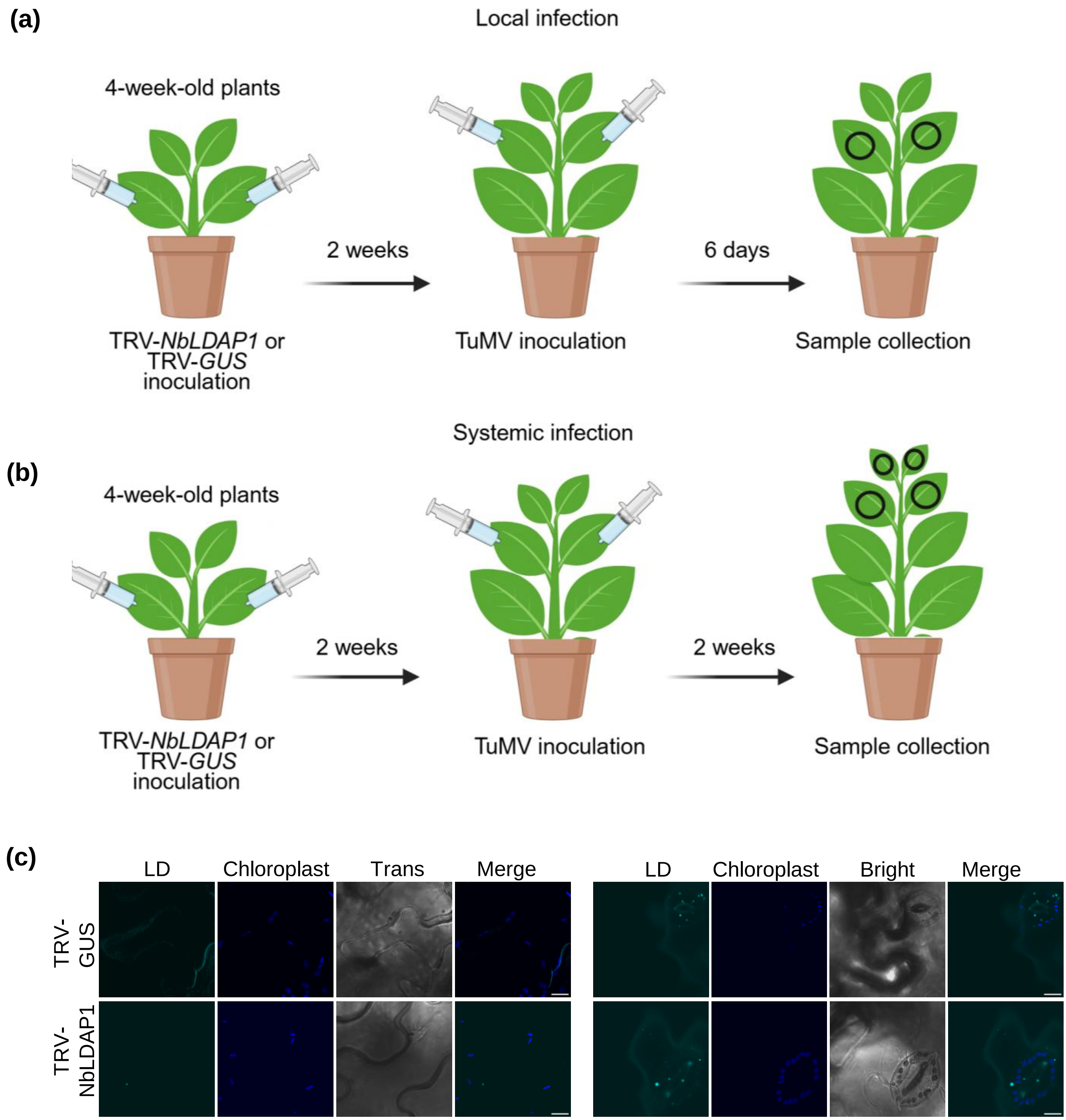

Fig. S3 Experimental design for local and systemic TuMV infection assays in *NbLDAP1*-silenced *Nicotiana benthamiana* plants and LD accumulation upon TRV infection carrying VIGS target constructs. (a) Experimental design for local TuMV infection assays. *A. tumefaciens* carrying the TRV-GUS or TRV-NbLDAP1 infectious clones were inoculated on 4-week-old *N. benthamiana* plants. Two weeks later, young leaves were infiltrated *with A. tumefaciens* carrying the TuMV-GFP infectious clone and leaf patches were collected at 6 days post-inoculation (dpi). **(b)** Experimental design for systemic TuMV infection assays. *A. tumefaciens* carrying the TRV-GUS, TRV-NbLDAP1 infectious clones were inoculated on 4-week-old *N. benthamiana* plants. Two weeks later, *A. tumefaciens* carrying the TuMV infectious clone were inoculated. The top three-four leaves were collected at 2 weeks post-inoculation. **(c)** Accumulation of LDs stained with MDH (cyan) upon TRV infection in the cells of systemically infected leaves. The right panel shows stomata in the epidermal cells that were used as positive control for MDH staining. Scale bar: 10 µm.

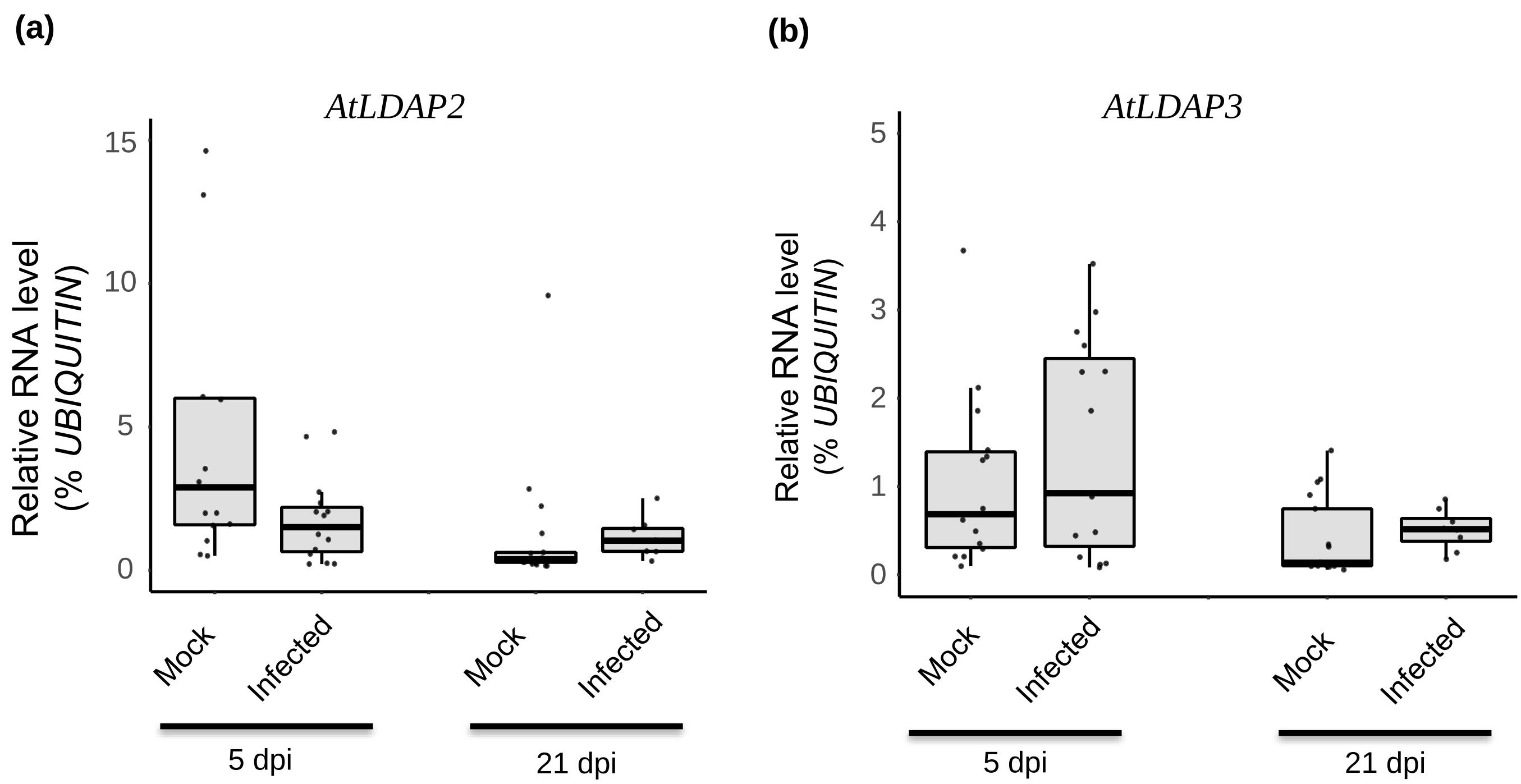

Fig. S4 ***AtLDAP2* (a) and *AtLDAP3* (b) expression levels do not change during TuMV infection.** Transcript levels measured by qRT-PCR were normalized to the expression of the *Ubiquitin* housekeeping gene in TuMV-GFP//mCherry inoculated and mock-inoculated *Arabidopsis* leaves at 5 dpi (inoculated leaves) and 21 dpi (systemically infected leaves). For each time point, gene expression was quantified from leaves sampled from 15 plants, and 3 independent experiments were performed. Thick horizontal lines within the boxes represent the median of the measurements; points indicate the value of each replicate. Statistical analysis was carried out using R software (Kruskal-Wallis test, not significant).

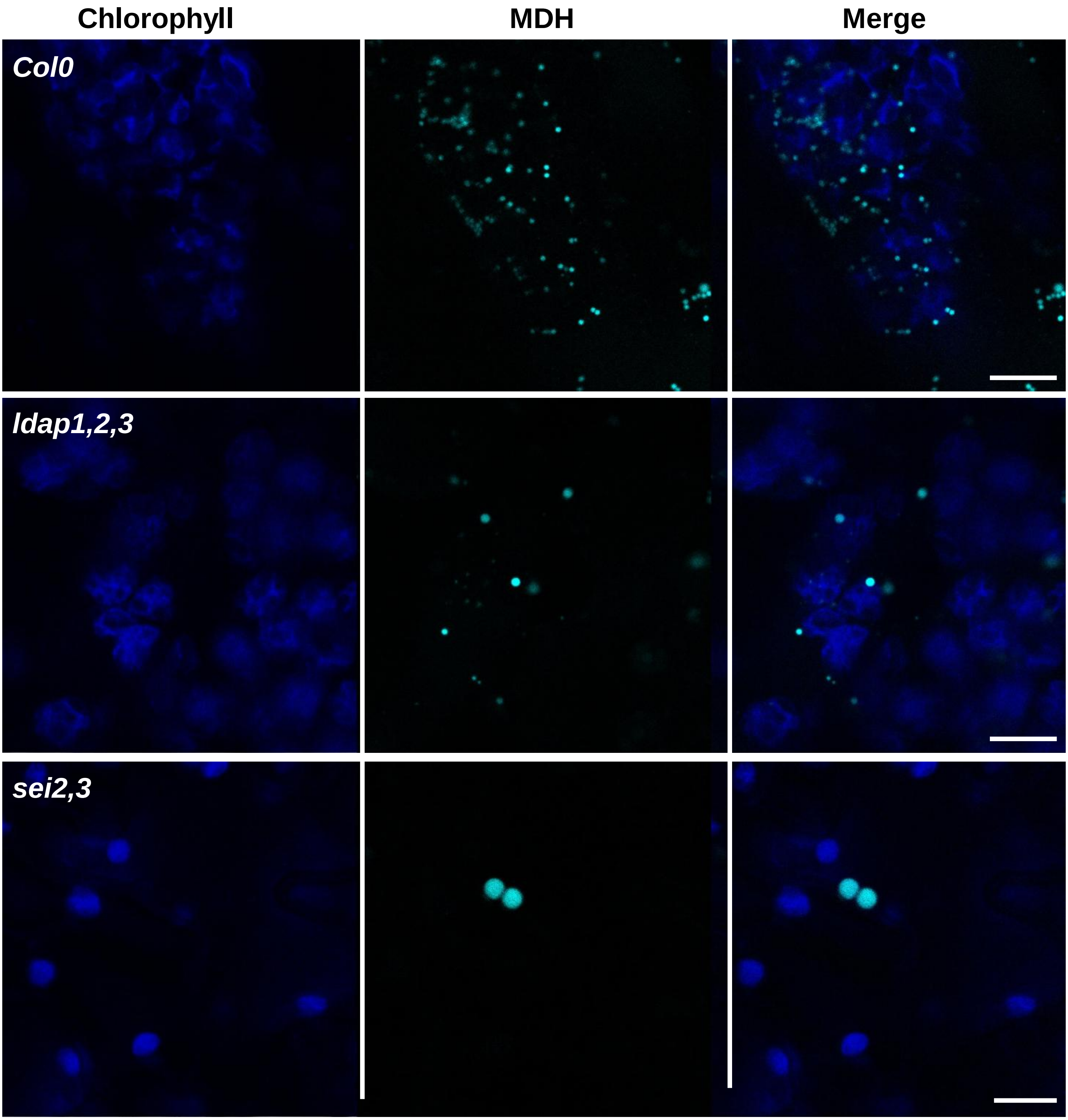

Fig. S5 ***ldap1,2,3*** **and** ***sei2,3*** **mutants accumulated fewer LDs compared to Col 0 after 7 days of nitrogen starvation, as observed by confocal microscopy.** Leaves were incubated in a solution of monodensylpentane (MDH) for LD staining before observation. In dark blue = chloroplasts and in cyan = LDs. Scale bar: 10 µm. Images are representative of 2 independent experiments and at least 4 plants per experiment.

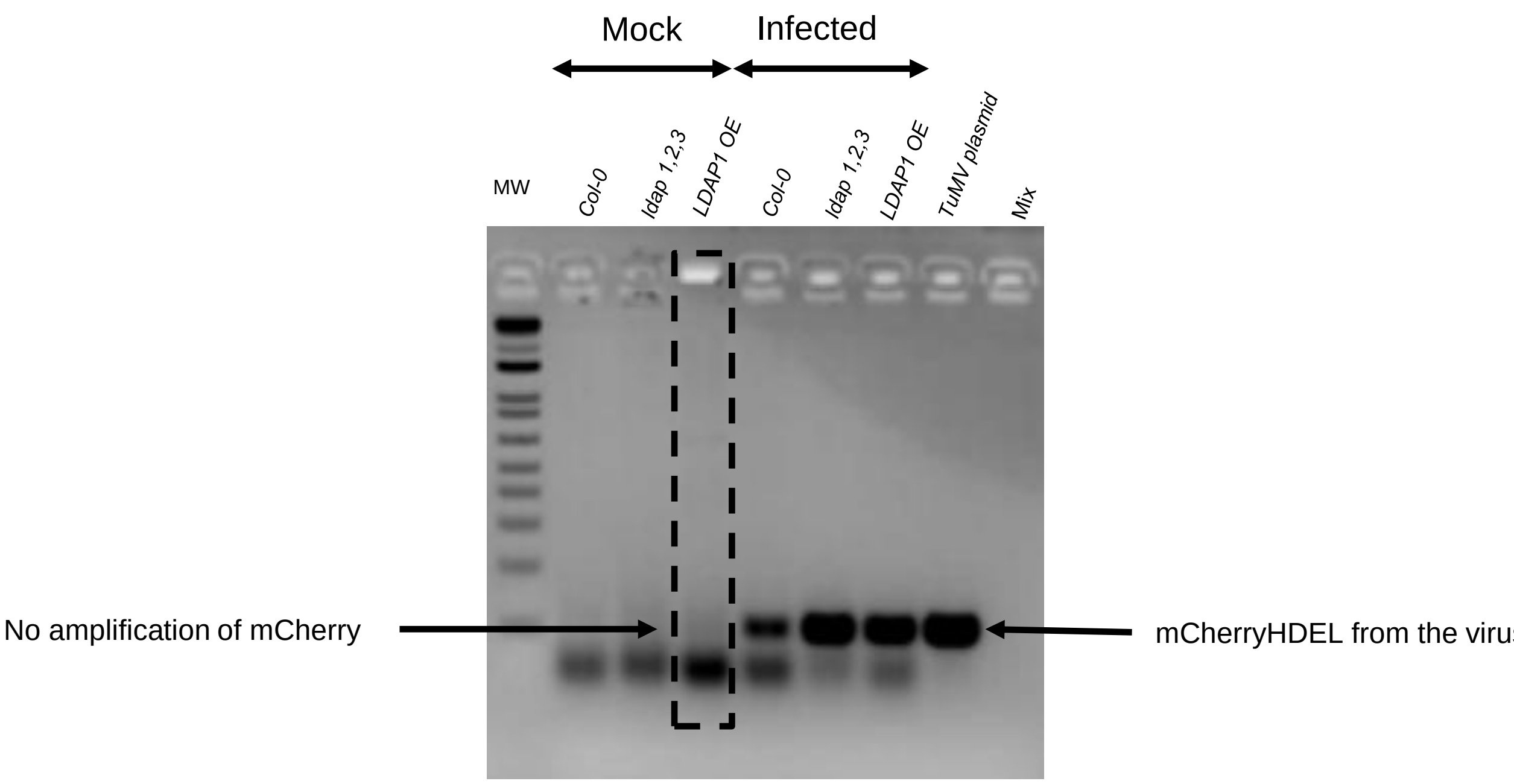

<u>Fig. S6</u> Control by RT-PCR of the specificity of primers designed to amplify the mCherryHDEL sequence (viral construct) but not the mCherry sequence present in the transgene in *LDAP1 OE* lines. RT-PCR amplification of *mCherry-HDEL* gene: no amplification was observed in the mock-inoculated plants, and especially in *OE-LDAP1* line (expressing a LDAP1-mCherry fusion) showing the specificity of the primers used.

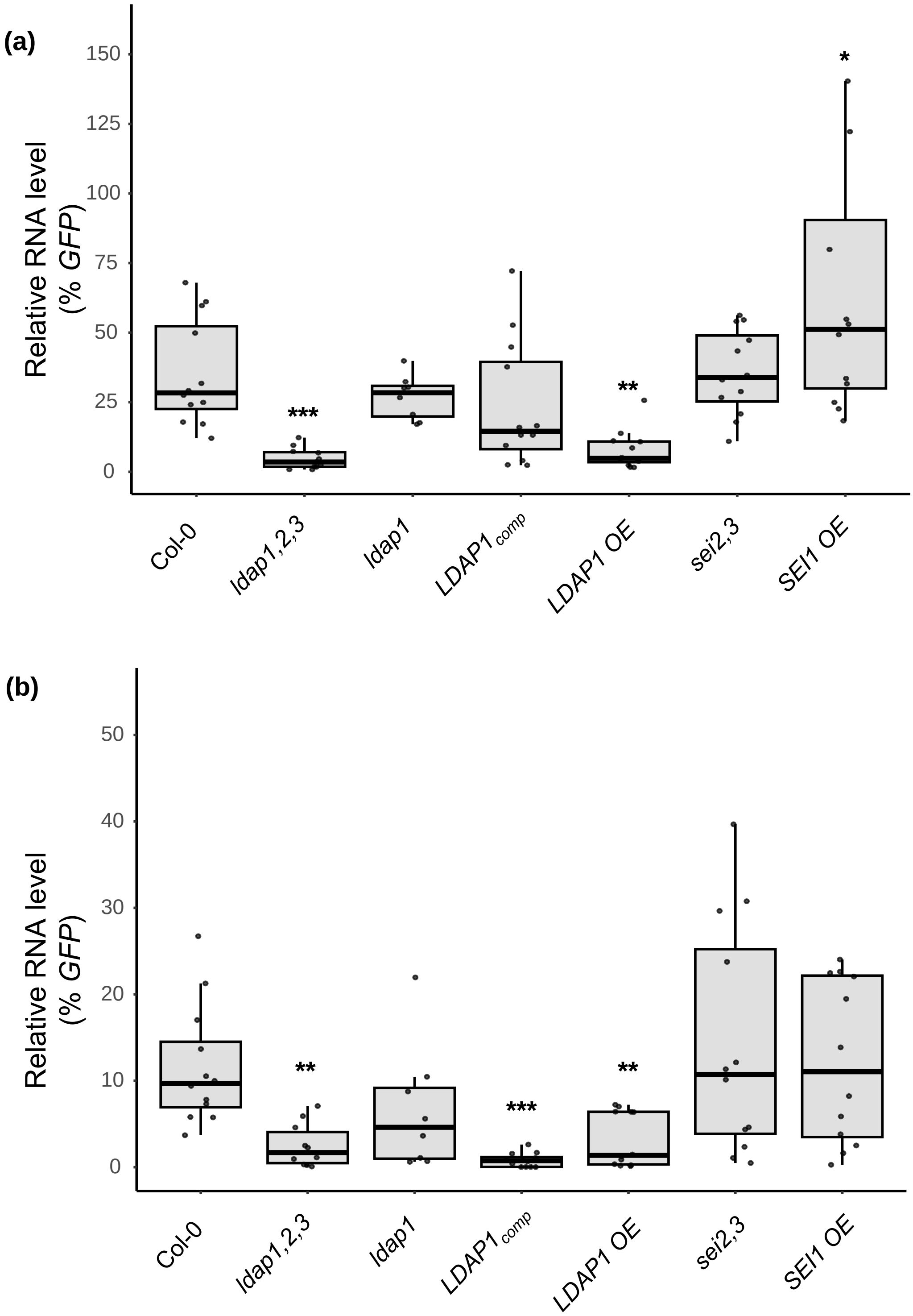

<u>Fig. S7</u> Evaluation of the accumulation of TuMV genomic RNA levels relative to GFP expression using the TuMV^{WT}6K2:mCherry//GFP (a) or TuMV^{W15A}6K2:mCherry//GFP (b) constructs in the wild-type (Col-0) and LD biogenesis related mutants by qRT-PCR. Gene expression was quantified in inoculated leaves sampled from 12 independent plants per genotype in 3 independent experiments at 5 dpi. Three technical Q-PCR replicates were performed for each sample. Normalization was done to GFP expression to correct for agroinfiltration efficiency ($2^{-\Delta(CtCP-CtUb)}$ / $2^{-\Delta(CtGFP-CtUb)}$). Note the reduced y-axis range in (b) due to the mutated 6K2 protein limiting viral movement. Thick horizontal lines within the boxes represent the median of the measurements; points indicate the value of each replicate. Statistical analysis was performed using a Kruskal-Wallis test followed by Dunn's test in R software (*$p < 0.05$, **$p < 0.01$, ***$p < 0.001$ compared to *Col-0*).

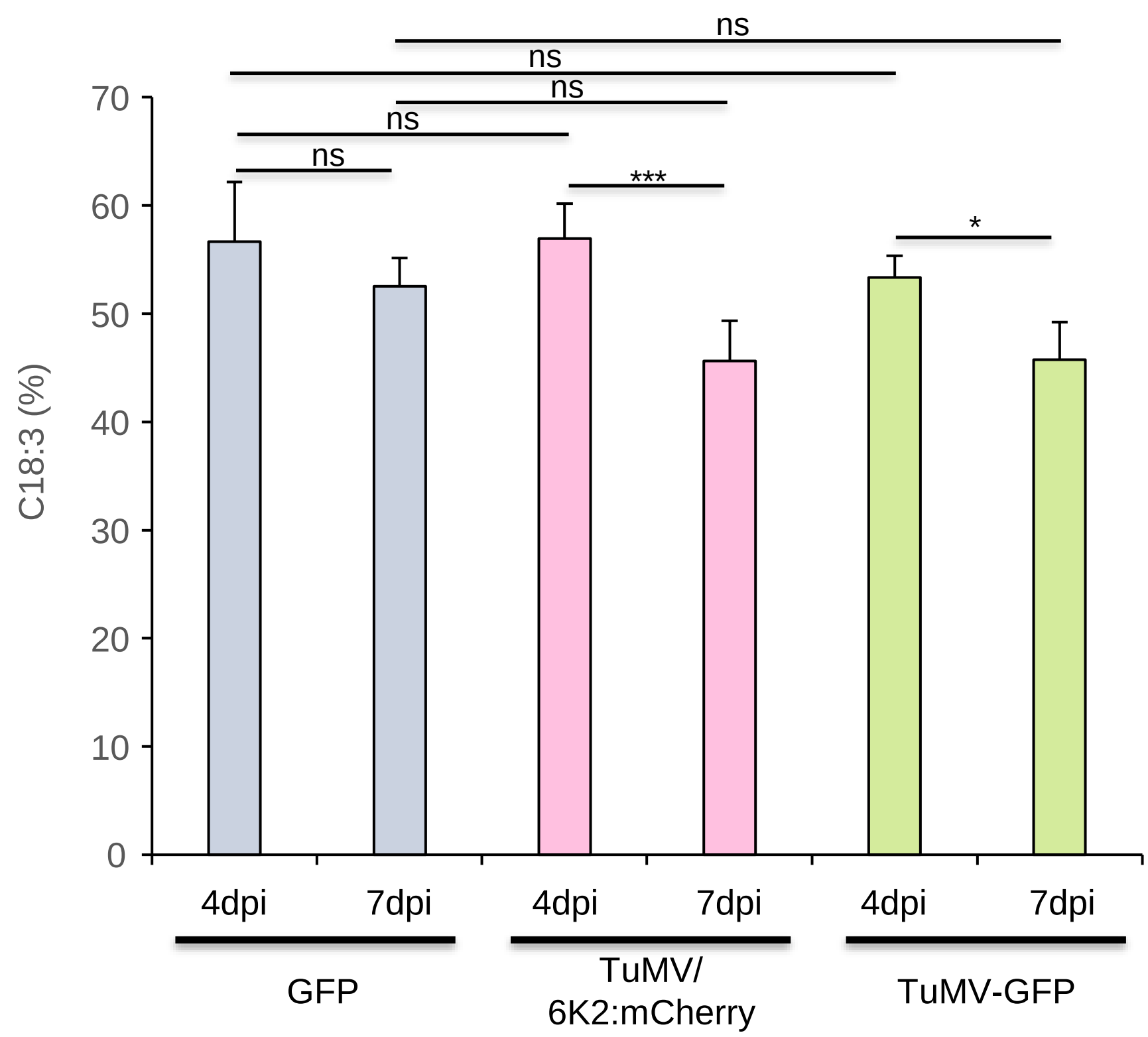

<u>Fig. S8</u> Analysis of C18:3 content in total lipids in TuMV- infected *N. benthamiana* leaves. *N. benthamiana* plants were inoculated with GFP (mock control), TuMV/6K2:mCherry or TuMV-GFP, and the C18:3 content in total lipids of the inoculated leaves (4 dpi) or in the systemically infected leaves (7 dpi) was determined by GC-FID. These data combined 12 biological replicates from two independent experiments. Error bars represent standard deviation. ns= non significant, * = $p<0.05$, *** = $p<0.001$ (Kruskall Wallis followed by Dunn's post-hoc).
